# Light Affects Host‐Symbiont Dynamics in the Non‐Photosynthetic Social Amoeba Symbiosis

**DOI:** 10.1002/ece3.71320

**Published:** 2025-04-18

**Authors:** Yuehui Tian, Lin Zhang, Zihe Wang, Zhili He, Longfei Shu

**Affiliations:** ^1^ School of Life Sciences Guangzhou University Guangzhou China; ^2^ School of Environmental Science and Engineering, Southern Marine Science and Engineering Guangdong Laboratory (Zhuhai) Guangdong Provincial Key Laboratory of Guangzhou Guangzhou China

**Keywords:** *Dictyostelium discoideum*, lectin genes, light‐induced symbiosis, *Paraburkholderia* species

## Abstract

Light significantly influences phototactic behaviors and host‐bacterial interactions of photosynthetic microorganisms such as algae. The non‐photosynthetic slime mound amoeba *Dictyostelium discoideum* as the host shows phototaxis in the multicellular slugs, but the impact of light on amoeba‐bacteria interactions remains unclear. Here we utilized two different clades of symbiotic *Paraburkholderia* species, namely *Paraburkholderia agricolaris* B1QS70 and *Paraburkholderia hayleyella* B2QS11, to investigate the light‐induced symbiosis between the host amoebae and symbiotic bacteria. Our findings propose two light‐induced symbiotic types (type I and type II termed from this study) likely due to amoebae metabolites or bacterial infection efficiency. The type I symbiosis reveals increased symbiotic B1QS70 amount in amoebae QS9 under light, while stable amounts persist in amoebae QS11 and QS70, both of which are native hosts of symbiotic *Paraburkholderia* species. Furthermore, the transcriptomics analysis suggests that certain upregulated genes, such as lectin genes, may play crucial roles in inducing the symbiosis of *P. agricolaris* B1QS70 in amoebae QS9 and QS70 under light stimulation. Conversely, the type II symbiosis enhances interactions between *P. hayleyella* B2QS11 and three individual amoebae clones (QS9, QS11, or QS70) in dark conditions due to the strong infection capability and high growth rates of B2QS11. Transcriptomic data show that a cluster of heat shock genes is upregulated in amoebae QS9 with B2QS11 under dark, indicating an immune response to the non‐native host QS9, rather than that of in QS11 as the native host of B2QS11. Blue‐light sensors like Cryptochrome/DNA photolyase in *Paraburkholderia* species might regulate the growth rate by light stimulation. These findings highlight light‐regulated symbiosis between amoebae and two distinct *Paraburkholderia* species, indicating that light may be crucial for regulating amoebae‐symbionts dynamics.

## Graphical abstract

This study highlights light‐regulated symbiosis between amoebae and two distinct Paraburkholderia species, indicating that light may be crucial for regulating amoebae‐symbiont dynamics.

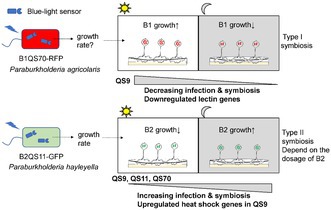

## 1 Introduction

Host‐bacterial interactions are prevalent in the majority of ecosystems. The symbiotic relationship between protists and prokaryotes can vary from parasitic to mutualistic status. Light may serve as a key driver in influencing the dynamics of host‐bacteria symbiosis. Phototaxis, observed in cyanobacteria, algae, and certain choanoflagellates, is one of the typical microbial behaviors that impact their physiological roles (Brunet et al. 2019; Menon Shakti et al. 2021; Ridge 2002). Photosynthetic microorganisms such as green algae and cyanobacteria can directly regulate their growth rates in response to different light conditions through photosystems. However, whether light can stimulate the interaction between non‐photosynthetic protists and bacteria is still largely unclear. *Dictyostelium discoideum*, as a social amoeba, performs a primitive farming symbiosis, including dispersal and prudent harvesting of bacteria (Brock et al. 2011). The development of *D. discoideum* includes unicellular and multicellular stages. In the unicellular stage, vegetative cells are germinated from spores and feed on bacteria by phagocytosis. Amoeba single cells then aggregate during starvation and form multicellular mounds and slugs, which further form fruiting bodies containing new amoeba spores for the next life cycle. The interaction between amoebae and *Paraburkholderia* species can be established throughout the amoebae life cycle, starting from the stage of amoebae vegetative cells by phagocytosis and extending to the stage of fruiting body formation. New spores will be dispersed from the fruiting body for a new life cycle. The establishment of bacteria symbiosis is facilitated by the chemotaxis of the host amoebae (Shu, Zhang, et al. 2018). The amoeba‐bacteria interaction will provide an ideal system for studying the light‐induced symbiosis (Shi et al. 2021).

It was reported in the last five decades that light can regulate the dynamic growth of *D. discoideum* NC‐4(H) and NC‐18 isolates (Konijn and Raper 1966; Reinhardt 1968). More aggregations and fruiting structures of amoebae NC‐4(H) were observed under constant light compared to those kept in the dark (Konijn and Raper 1966). Phototaxis behaviors are observed in amoebae at specific developmental stages, such as the migration of multicellular slugs towards a light source due to the local release of cAMP (Miura and Siegert 2000). The oscillations of cytosolic cAMP can induce a transition in amoebae single cells from quiescence to rhythmic activity, playing a key role in the collective behavior (Gregor et al. 2010). In addition, it was reported that some cytosolic proteins like RasD, FLN can also regulate the phototactic response of amoebae slugs (Khaire et al. 2007; Wilkins et al. 2000). However, whether amoebae have upstream photoreceptors like membrane‐bound rhodopsins is still unclear. The study of light‐induced symbiosis between amoeba *Dictyostelium discoideum* and *Paraburkholderia* will provide new insights to unravel mechanisms of host‐bacteria interactions. *Paraburkholderia* were divided into two clades, B1 (*Paraburkholderia agricolaris*) and B2 (*Paraburkholderia hayleyella*), with quite different genome sizes isolated from amoebae (DiSalvo et al. 2015). This distinction suggests that these two *Paraburkholderia* species may have distinct types of symbiotic relationships with amoebae. Notably, compared to other strains, these two *Paraburkholderia* species can form stable symbiotic associations with *D. discoideum* (Haselkorn et al. 2019; Shu, Brock, et al. 2018). By comparing different types of symbiosis, we can dissect the molecular mechanisms underlying host‐bacteria interactions.

Some microorganisms with or without photosynthetic components are capable of responding to light through various photoreceptors. For example, some green algae, fungi, soil bacteria, or protists possess photoreceptors such as Cyclops (cyclase opsins), bPAC (photoactivated adenylyl cyclase), or phytochrome, which are stimulated by different wavelengths of blue, green, and even red light (Auldridge and Forest 2011; Avelar et al. 2014; Gao et al. 2015; Stierl et al. 2011; Tian et al. 2018, 2021). In these microorganisms, light illuminations may obviously influence physiological processes like the phototaxis of *Chlamydomonas* and *Blastocladiella* or light‐regulated colony inversion in a multicellular *Choanoeca flexa* (Brunet et al. 2019). This observation provides valuable insights into understanding how light serves as a driving force in inducing the growth of amoebae and bacteria, as well as regulating the symbiotic relationship between host and bacteria.

In this study, we detected the fluorescence to depict the growth rate of both *Paraburkholderia* species B1QS70 and B2QS11 with individual fluorescence RFP or GFP tag labeling. Furthermore, we also examined the fluorescence of a certain number of single amoebae cells and newly germinated spores carrying symbiotic bacteria under varying durations of light exposure. We observed that each symbiosis type exhibited similar patterns, ranging from unicellular amoebae cells in the exponential stage to newly germinated spores. The type I symbiosis between *P. agricolaris* B1QS70 and *D. discoideum* QS9 was enhanced directly by light, while the symbiosis was largely reduced under dark conditions. The transcriptomic analysis revealed that the upregulated lectin genes may play essential roles in host defense by resisting bacteria, thereby attenuating digestion or degradation of bacteria under light treatment. In comparison, the type II symbiosis between *P. hayleyella* B2QS11 and three amoeba clones QS9, QS11, QS70 suggested that the increased growth rate of B2QS11 under dark conditions tended to promote the infection and symbiosis with amoebae. The upregulated heat‐shock genes in amoebae under dark conditions could cause immune responses in *D. discoideum* QS9, rather than in the native host QS11 with symbiotic *P. hayleyella* B2QS11. We speculate that blue‐light sensors could induce the growth conditions of *Paraburkholderia* species and trigger different types of symbiosis. This study provides a new possibility or evidence to unveil the light‐driven force as one of the essential elements influencing the host‐bacteria symbiosis during evolution.

## 2 Materials and Methods

### 2.1 Amoebae Clones and *Paraburkholderia* Culture Conditions

In the symbiosis study, three *D. discoideum* isolates (QS9, QS11, QS70) were selected to mix with individual *Paraburkholderia* species B1QS70, B2QS11. QS9 has no symbiotic bacteria. *Paraburkholderia* B2QS11 and B1QS70 in their natural hosts QS11 and QS70 were removed by antibiotics (Shu, Brock, et al. 2018). This enables us to recombine individual hosts and one *Paraburkholderia* species in two groups of this study as below: QS9 + B1QS70, QS11 + B1QS70, QS70 + B1QS70; QS9 + B2QS11, QS11 + B2QS11, QS70 + B2QS11. For all the culture plates, 2 × 10^5^ *D. discoideum* spores were grown on SM/5 agar plates and mixed with feeding bacteria *K. pneumoniae* (OD_600_ = 1.5, 200 μL/each plate). KK2 buffer was used for dilution or transfer of amoebae spores or bacteria (2.25 g KH_2_PO_4_ and 0.67 g K_2_HPO_4_ per liter). The ingredients of SM/5 agar plates include 2.0 g of yeast extract (Oxoid), 2.0 g BactoPeptone (Oxoid), 2.0 g glucose, 1.9 g KH_2_PO_4_, 1.0 g K_2_HPO_4_, 0.2 g MgSO_4_, and 15.0 g agar per liter. The *Paraburkholderia agricolaris* B1QS70 and *Paraburkholderia hayleyella* B2QS11 strains were also grown on SM/5 agar plates. To reestablish the symbiotic *Paraburkholderia* species in amoebae, an additional 10 μL (OD_600_ = 1.5) *P. agricolaris or P. hayleyella* was mixed with amoebae and *K. pneumoniae* as above in each plate. For the light or dark treatment, some plates were put at 21°C in a light incubator as the normal control group, and others were covered by foil papers to mimic dark treatment in the same incubator as the treatment group. The same batch of amoebae spores and bacteria were used in each experiment.

### 2.2 Detection of Fluorescence From Bacteria and Amoebae Cells

In this study, the two *Paraburkholderia agricolaris* B1QS70 and *Paraburkholderia hayleyella* B2QS11 strains were stably labeled by RFP and GFP tags respectively (DiSalvo et al. 2015). To detect the fluorescence of symbiotic bacteria in amoebae vegetative cells after 36 h incubation, approximately 2 mL ice precooling KK2 buffer was used to wash out the amoebae cells mixed with bacteria in agar plates. Then the mixture was centrifuged at 1000*g*, 4°C for 3 min, and the supernatant was discarded with extracellular bacteria. The washing step was repeated three times until the extracellular bacteria were removed. The pellet was then resuspended in ~300 μL KK2 buffer. Fluorescence emission values of 200 μL resuspended buffer with amoebae cells were then detected by a microplate fluorometer (Thermo Scientific Fluoroskan). The fluorescence of GFP was detected by using an excitation wavelength of 485 nm and an emission wavelength of 515 nm. Then the fluorescence of RFP was measured by using an excitation wavelength of 555 nm and an emission wavelength of 585 nm. The remaining resuspended buffer with amoebae was diluted in KK2 buffer and amoebae cells were counted on a hemocytometer. The total number of amoebae cells was calculated back to the 200 μL resuspended buffer. Therefore, the fluorescence from a certain number of amoebae cells (normalized to 1 × 10^7^ amoebae cells or as indicated) was confirmed. Similarly, in the fruiting body stage, the amoebae spores were harvested from plates after 7 days incubation. The washing steps were performed as above. Finally, the fluorescence from a certain number of amoebae spores (normalized to 1 × 10^7^ amoebae spores) was confirmed. For the fluorescence measurement of *Paraburkholderia* species, the 12‐well plate with SM/5 agar was used to mimic the growth condition of bacteria with amoebae. The original OD600 was designed to be around 0.1. The incubation time points were selected at 0 h, 9 h, 27 h, 36 h, and 48 h. The bacterial plaque in each individual well was washed out with ~200 μL KK2 buffer and the fluorescence was detected directly by the microplate fluorometer. For all the fluorescence tests as described above, we operated at least three biological replications in each experiment. This biological replication information is shown in figure legends as indicated.

### 2.3 Spore Production Assays

When comparing total spore numbers in each plate under dark and light, 2 × 10^5^ *D. discoideum* spores QS9, QS11, and QS70 were separately grown on SM/5 agar plates with 200 μL *K. pneumoniae* (OD_600_ = 1.5). Symbiosis was established by further adding 10 μL (OD_600_ = 1.5) *P. agricolaris or P. hayleyella* to the above mixture. The dark and light incubation time was determined 7 days after plating. We used approximately 5 mL KK2 buffer with 0.1% Nonidet P‐40 to wash out the fruiting body from the plate to harvest spores. All the samples were diluted 10 times and counted on a hemocytometer. We employed three biological replicates in each group under dark and light. Three groups were tested as below: non‐symbiotic amoebae QS9, QS11, and QS70 (light, dark treatment), recombined symbiotic amoebae QS9 + B1QS70, QS11 + B1QS70, and QS70 + B1QS70 (light, dark treatment), and recombined symbiotic amoebae QS9 + B2QS11, QS11 + B2QS11, and QS70 + B2QS11 (light, dark treatment).

### 2.4 Microscopy

Fluorescent images were acquired by Echo Revolve fluorescent microscope at ×40 or ×200 magnification as indicated. The fluorescence of amoebae with symbiotic B2QS11‐GFP was detected when excited by blue light at ~485 nm and detected at ~515 nm in the microscope. The fluorescence of amoebae cells with symbiotic B1QS70‐RFP was detected when excited by green light at ~532 nm and detected at ~580 nm. Amoebae vegetative cells were taken after 36 h incubation with *K. pneumoniae* and individual *Paraburkholderia* species. Amoebae cells were washed three times as above centrifuged steps to remove the extracellular bacteria. This allows us to detect the symbiotic bacteria more clearly. After the centrifugation, the pellet was resuspended in ~1 mL KK2 buffer. Then 10 μL suspension with amoebae cells was used for imaging with a ×20 objective (×200 total magnification) on the Echo Revolve fluorescent microscope. The amoebae spores were taken from the fruiting body and imaged as the same processes mentioned above. The fruiting body of amoebae was directly imaged from the individual plates by using a ×4 objective (×40 total magnification) on the fluorescent microscope. Host‐symbiont pairs were imaged after acclimation for 2–3 social cycles. Here we used ZEISS LSM 980 Laser Scanning Microscope to take confocal images. To detect RFP fluorescence, the excitation wavelength and emission window settings were designed at 543 nm and 570–670 nm. To detect GFP fluorescence, the excitation wavelength and emission window settings were designed at 488 nm and 500–530 nm.

### 2.5 Statistical Analysis of Fluorescence Tests

The GraphPad Prism 8 software package was employed to do statistical analyses. All the fluorescence measurements from the fluorophotometer were designed with at least three biological replicates. The results are shown with mean values and standard deviations (SD) in the figure legends. Data from fluorescence between light and dark treatment were analyzed using an unpaired two‐tailed Student's t test. For multiple comparison among different treatment time points, multiple t‐test between identical groups in each time point was performed. Statistical analyses were also performed using ANOVA with Tukey's multiple‐comparison test as indicated in the figure legends. *p* values were determined as statistical significance (* indicates *p* < 0.05; ** indicates *p* < 0.01; *** indicates *p* < 0.001; and **** indicates *p* < 0.0001).

### 2.6 Transcriptome Sequencing and Bioinformatic Analysis

Two test groups were designed for transcriptomic analysis. In the first group, amoebae QS9 and QS70 vegetative cells (~1 × 10^7^) with symbiotic B1QS70‐RFP were harvested at 36 h under constant dark and light treatment, respectively. In the second group, amoebae QS9 and QS11 vegetative cells (~1 × 10^7^) with symbiotic B2QS11‐GFP were harvested at 36 h under dark and light treatment. Three biological replications were performed under light as the control and dark as the treatment, respectively. The extracellular bacteria were removed by three times centrifugation steps as indicated washing processes above. All the cell collection steps were performed on ice. The cell pellets were resuspended by the TRIzol reagent (Invitrogen, Carlsad, CA, USA), and RNA extraction was performed afterwards. The light condition was designed as the control group, while the dark incubation was regarded as the treatment group. Therefore, we compared the different gene expression patterns of *D. discoideum* cells with symbiotic *Paraburkholderia* species in dark and light. Sequencing libraries were generated by using an Illumina Novaseq 6000 platform (Illumina, CA, USA) at Majorbio Technology Co. Ltd. (Shanghai, China). *D. discoideum* reference genome (GCF_000004695.1) was used to align to the trimmed and clean data via Bowtie2 v2.4.1. Differential expressed genes (DEGs) were identified by DESeq2 Version 1.24.0 with a fold change more than 2.0 (*p* < 0.05). DEGs were drawn in heatmaps with log_10_ (TPM + 1) and Volcano Plots with *x*‐axis log_2_ (S1/S2), and S2 indicates the control group. Goatools was used to finish gene ontology (GO) enrichment analysis in the DEGs. GO terms were regarded significantly enriched with corrected *p* value less than 0.05.

## 3 Results

### 3.1 Two Types of Light‐Regulated Amoebae‐Bacteria Symbiosis

**FIGURE 1:**
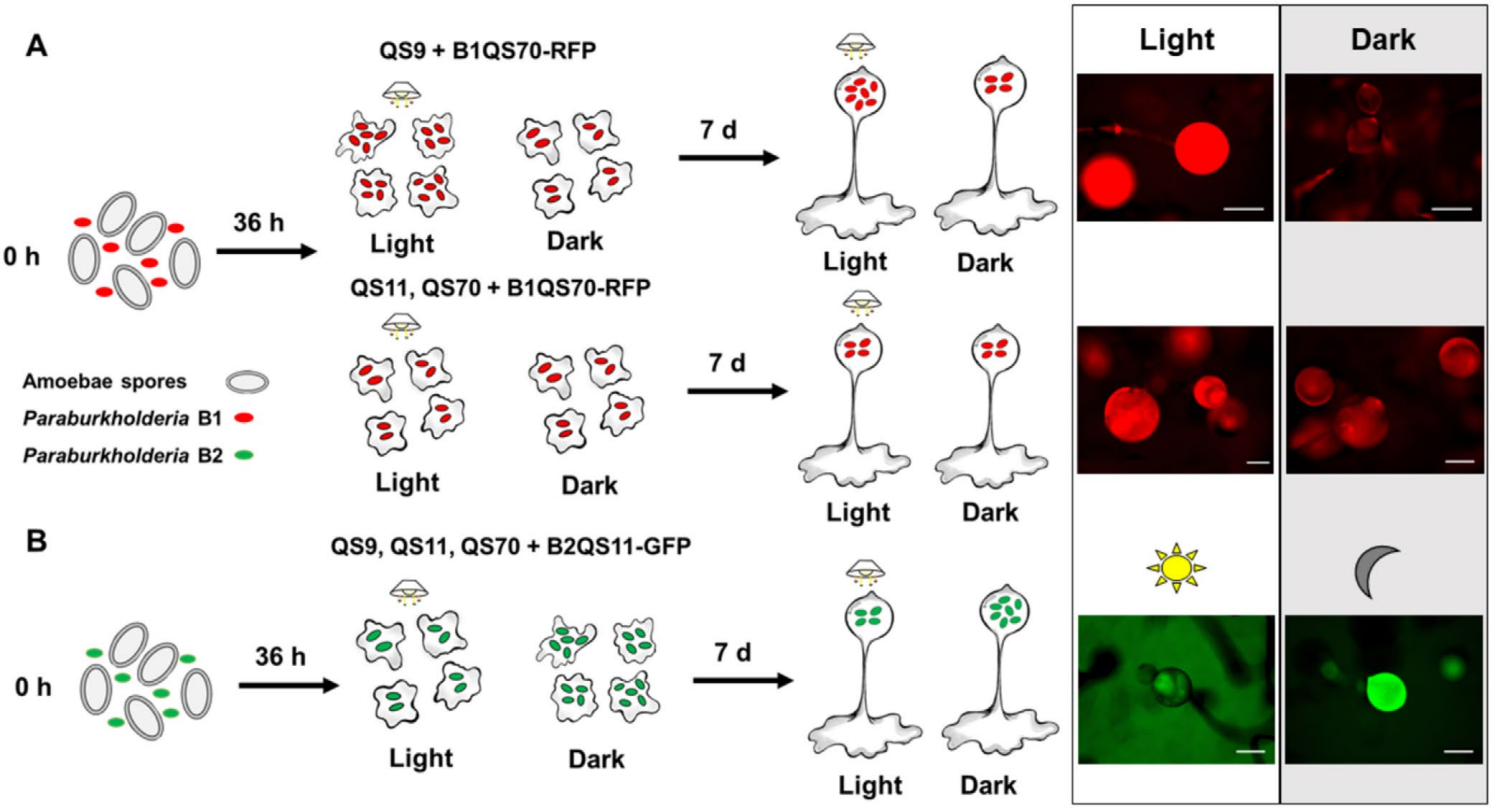
Schematic model of two types of symbiosis between *Paraburkholderia* B1QS70 or B2QS11 bacteria and amoebae under light stimulation (A, B). The symbiosis between RFP labeled B1QS70 *Paraburkholderia* and *Dictyostelium discoideum* (A). The symbiosis between GFP labeled B2QS11 *Paraburkholderia* and *Dictyostelium discoideum (B)*. For (A) and (B), the fluorescence was detected at the single cellular stage after 36 h of treatment and at the fruiting body stage after 7‐day incubation. Scale bar = 300 μm.

We conducted an experiment involving three distinct amoebae clones, namely QS9, QS11, and QS70, which were combined with feeding bacteria *Klebsiella pneumoniae* and symbiotic *Paraburkholderia* species (B1QS70, B2QS11). Following treatment for either 36 h or 7 days, we examined the symbiosis states by measuring fluorescence in both single‐cell vegetative amoebae and newly germinated spores within the fruiting body (Figure 1). Through this experimental model, we observed that the interactions between the host and bacteria were susceptible to variations in lighting conditions, yielding different outcomes for each specific combination.

*Paraburkholderia* species utilized in this study on light‐induced symbiosis were *P. agricolaris* B1QS70, labeled with RFP tag (B1QS70‐RFP) and *P. hayleyella* B2QS11, labeled with GFP tag (B2QS11‐GFP). The fluorescence emitted by these bacteria can be directly detected and reflects their dynamic growth rates during incubation (Figure S1). It is obvious that the growth rate of *P. agricolaris* B1QS70 remains consistent under dark and blue light at various time points, ranging from 9 to 48 h. However, the growth rate of *P. hayleyella* B2QS11 was obviously inhibited after blue light stimulation for 27 h (Figure S1). The fluorescence of *Paraburkholderia* within amoebae can also be measured from certain amounts of amoebae cells at the exponential stage, as well as newly germinated spores from the fruiting body (Figure S2). It is obvious that the fluorescence becomes linearized when using over 2 × 10^6^ amoebae spores from the fruiting body with either symbiotic B1QS70‐RFP or B2QS11‐GFP (Figure S2). This enables us to dynamically study the symbiosis of *Paraburkholderia* in amoebae under different light conditions.

In this study, we made an interesting observation regarding the influence of light on the dynamics between the host and symbiont. Specifically, we found that constant exposure to light during the incubation period led to the enhanced growth of intracellular B1QS70‐RFP in both unicellular (36 h) and multicellular fruiting body stages (7 days) when mixed with non‐symbiotic QS9 amoebae spores (Figure 1A). We have termed this type of symbiosis type I. However, the cured QS11 and QS70 amoebae (as natural symbiotic hosts) without intracellular *Paraburkholderia* species were detected with similar fluorescence at both 36 h and 7 days under light and dark after these amoebae were mixed with B1QS70‐RFP. This indicates light illumination plays a key role in enhancing the interactions between non‐symbiotic amoebae QS9 and B1QS70. Whereas, the symbiosis between natural symbiotic amoebae QS11, QS70, and *P. agricolaris* B1QS70 remains stable under dark and light conditions (Figure 1A). Furthermore, it is worth noting that this stable symbiosis status can be maintained from unicellular vegetative cells (36 h) to newly germinated amoebae spores (7 days).

However, a different type of symbiosis (type II symbiosis) was observed between individual amoebae QS9, QS11, or QS70 and *P. hayleyella* B2QS11‐GFP in a light‐controlled manner. Interestingly, we observed that the fluorescence of amoebae was stronger in both unicellular cells (36 h) and germinated spores (7 days) when they were mixed with B2QS11‐GFP under dark compared to light conditions (Figure 1B). This indicates that amoebae‐bacteria interactions can be increased in the absence of light. Furthermore, this symbiotic relationship appears to persist from single cellular to newly germinated amoebae spores.

### 3.2 Symbiosis of *P. agricolaris* B1QS70 in Amoeba QS9 Vegetative Cells and Spores Is Enhanced Under Light Illumination but Stabilized in QS11 and QS70

**FIGURE 2:**
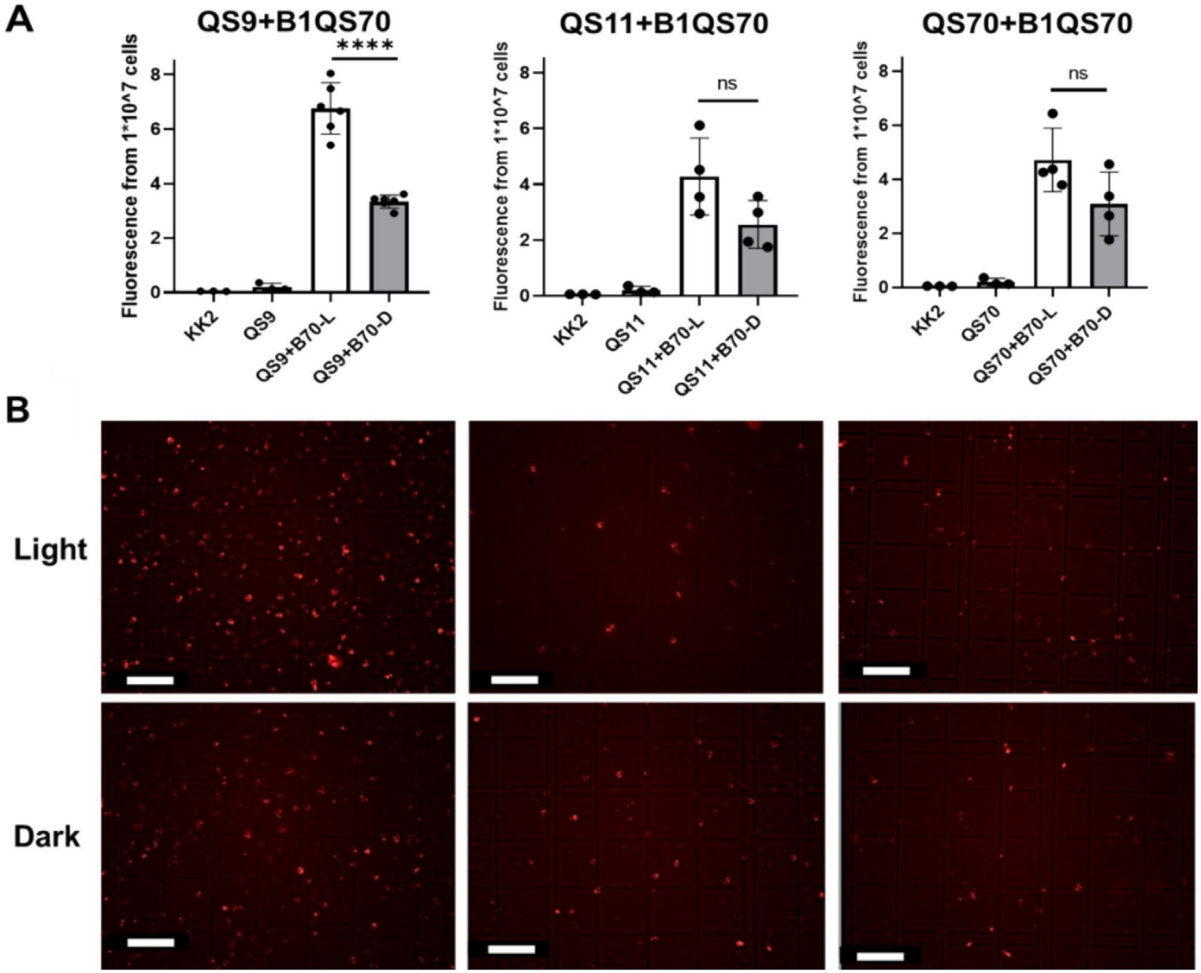
Fluorescence of amoeba cells at the exponential stage (incubated with *Paraburkholderia* B1QS70 for 36 h). (A) The fluorescence of a certain amount of amoebae cells (QS9, QS11, QS70, 1 × 10^7^) with symbiotic B1QS70 was detected under both dark and light conditions. KK2 and QS9, QS11, QS70 without symbiotic bacteria represent the blank and negative controls. Statistical significance was determined using Student's *t*‐test. Data are mean ± SD of 3–6 independent experiments (****p < 0.0001; ns indicates no significance). (B) The fluorescence images of amoebae QS9, QS11, QS70 with symbiotic B1QS70 were taken under light and dark (scale bar = 40 μm).

From the fluorescence detection, we observed that the growth of *P. agricolaris* B1QS70 was similar under both dark and light conditions (Figure S1A). The dynamic changes of symbiotic B1QS70 bacteria under light conditions could be largely dependent on the growth status of amoebae. When we compared the total number of geminated spores under dark and light, it was evident that the total number of spores on individual plates under light was approximately 3–5 times higher than that in the dark (Figure S3). This phenomenon was observed not only in the non‐symbiotic amoebae QS9, QS11, and QS70, but also in the B1QS70 and B2QS11 symbiotic amoebae. In the native QS9 clone mixed with *P. agricolaris* B1QS70, the fluorescence within amoebae cells (1 × 10^7^) was significantly higher (two times) in light than in the dark after 36 h of incubation (Figure 2A). The same number of QS9 vegetative cells as the negative control shows less fluorescence, similar to buffer KK2. The amoebae QS11 and QS70 used in this study have been cured by antibiotics to remove their native symbiotic *P. hayleyella* B2QS11 and *P. agricolaris* B1QS70, respectively. When B1QS70‐RFP was mixed with QS11 and QS70 to reestablish the symbiosis status, it was surprising to observe no significant difference of fluorescence in amoebae QS11 and QS70 under both light and dark treatment, although the fluorescence in the dark exhibited a trend towards being lower than that in the light (Figure 2A,B). This suggests that light‐enhanced *P. agricolaris* B1QS70 symbiosis in amoebae QS9 is not applicable to both amoebae QS11 and QS70, which originally have their native symbiotic *Paraburkholderia* species. Therefore, we suggested that the type I light‐induced symbiosis between amoeba QS9 and *P. agricolaris* B1QS70 could be enhanced and established initially, whereas the reestablished host amoebae QS11/QS70 with symbiotic bacteria cannot be influenced significantly by light possibly due to symbiont accompanied adaptation to the host.

To compare the dynamics between new exposure to symbiosis (*P. agricolaris* B1QS70 in amoeba QS9) and an established symbiosis (*P. agricolaris* B1QS70 in amoeba QS70), we further investigated type I symbiosis after acclimation for three social cycles. In comparing the fluorescence of amoeba QS9 with symbiotic B1QS70‐RFP over the three social cycles, we found that the symbiont B1QS70 in QS9 was enhanced under light illumination compared to the dark treatment (Figure S4). This indicates that light‐induced type I symbiosis could be gradually established during the acclimation period between host and symbiont. Meanwhile, we assessed the fluorescence of amoebae QS70 with symbiotic B1QS70‐RFP across the three social cycles. The results indicated that the fluorescence levels of symbiont B1QS70 in QS70 were similar in the first and second social cycles under both light and dark conditions (Figure S5). However, in the third social cycle, we observed a stronger fluorescence under light compared to dark conditions. This suggests that a trend of light‐enhanced fluorescence of symbiont B1QS70 in QS70 may still be observed in certain social cycles (Figure S5). Thus, it appears that the symbiont B1QS70 in amoebae QS70 may not have established stably, and the light‐induced type I symbiosis could be observed in certain social cycles of QS70 with symbiont B1QS70 during the acclimation period for establishing a stable symbiosis. Similarly, the new germinated spores from the fruiting body, after 7 days incubation, still maintain the same symbiosis type (Figure S6) as the vegetative amoebae cells at 36 h. The fluorescence emission of spores in the fruiting body was detected to compare the symbiosis status under light and dark incubation. In the *P. agricolaris* B1QS70 symbiosis of amoebae QS9, the data showed that the fluorescence of spores in constant light is significantly higher than that in constant dark. Furthermore, the symbiosis remains similar to constant light when transferring samples from light (for 5 days) to dark (for another 2 days) (L5D2), or vice versa (D5L2) in the dark (Figure S6A). The negative control of QS9 spores without *P. agricolaris* B1QS70‐RFP was detected to emit quite low fluorescence, similar to buffer KK2. In the symbiosis between *P. agricolaris* B1QS70‐RFP and amoebae QS11/QS70, the results suggested that no significant difference of fluorescence between amoebae QS11 and QS70 was observed under light/dark treatments (Figure S6A). Furthermore, the fluorescent images showed that light can dramatically increase the *P. agricolaris* B1QS70 symbiosis only in QS9, but not in QS11 and QS70 amoebae fruiting body (Figure S6B). The fluorescence of QS9 spores from 5 days of light incubation and 2 days of dark condition (L5D2) is similar to constant light illumination for 7 days. Moreover, the fluorescence of QS9 spores from 5 days of dark treatment and 2 days of light condition (D5L2) is similar to constant dark condition for 7 days (Figure S7). The light‐enhanced *P. agricolaris* B1QS70 symbiosis in amoebae QS9 spores at 7 days is still similar to the symbiosis status of vegetative cells at 36 h. This indicates that the symbiosis status between amoebae and *P. agricolaris* B1QS70 remains consistent from the unicellular stage to the fruiting body stage.

In addition, we investigated other fluorescently tagged *Paraburkholderia agricolaris* strains, specifically B1QS159‐RFP and B1QS161‐RFP, to assess light‐induced symbiosis. Here we reestablished symbiosis between each amoeba (QS9, QS11 or QS70) and the corresponding fluorescently tagged B1QS159 or B1QS161. The data showed that the symbiont B1QS159‐RFP or B1QS161‐RFP in amoeba QS9 is also enhanced under light (Figure S8A,B). In contrast, the symbiosis of B1QS159‐RFP and B1QS161‐RFP in amoebae QS11 (Figure S9) or QS70 (Figure S10) appeared to be more stable under both dark and light conditions. Although we observed a reduced trend in symbiosis under dark conditions for QS11 and QS70 with the two *P. agricolaris* strains, the fluorescence changes of the symbiont in these amoebae were not as obvious as those observed in QS9 with the same *P. agricolaris* strains.

### 3.3 Transcriptome Profiles of QS9 and QS70 With *P. agricolaris* B1QS70 at the Exponential Stage Under Light and Dark

**FIGURE 3:**
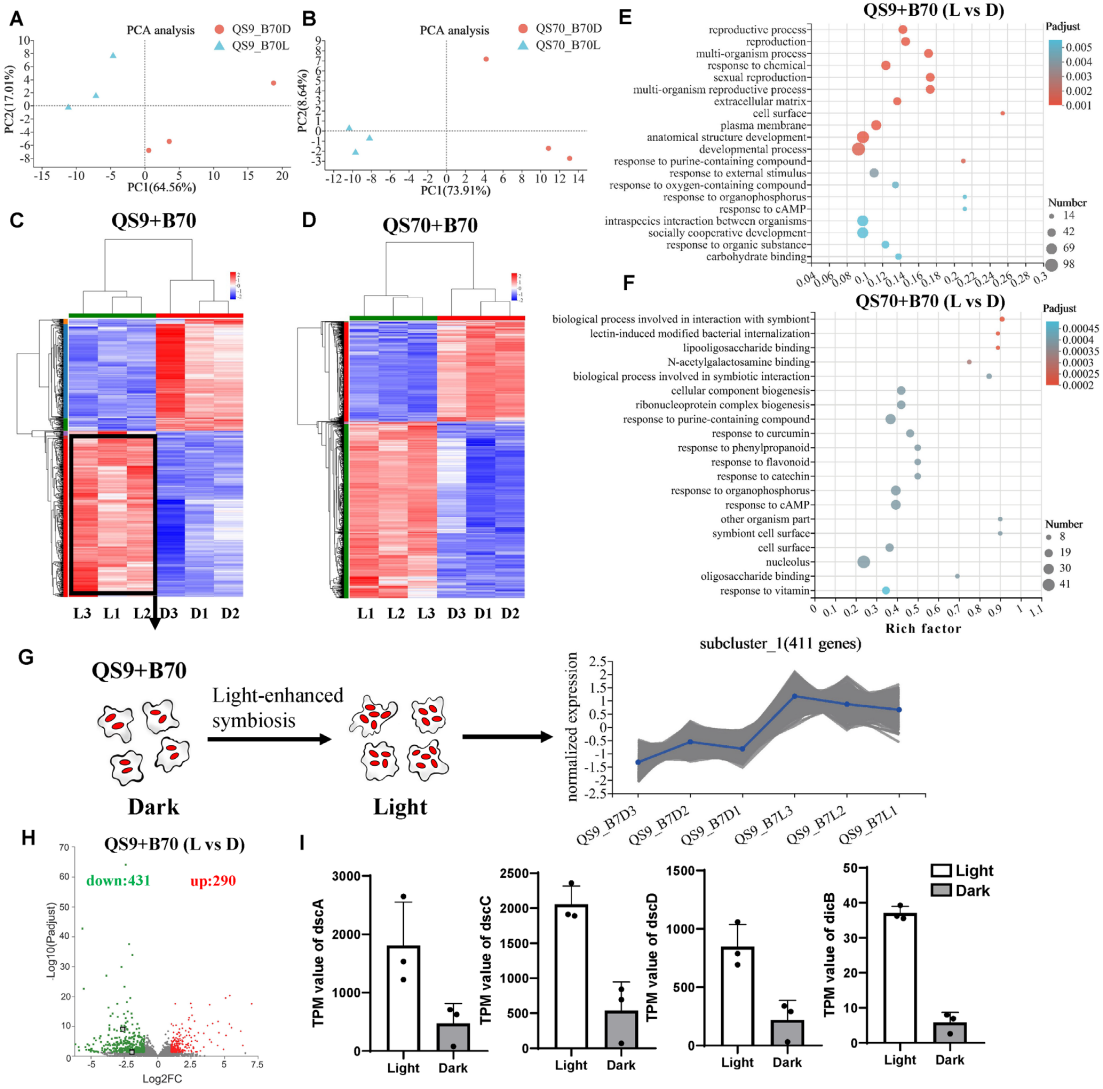
Transcriptome profiles of QS9 and QS70 amoebae with B1QS70 after 36 h of dark and light incubation. Three biological replications were performed under light as the control and dark as the treatment, respectively. Principal component analysis (PCA) was performed on RNA‐seq datasets from amoebae QS9 (A) and QS70 cells (B), respectively, in symbiosis with B1QS70 under light and dark conditions. Heatmaps of differentially expressed genes (DEGs) from QS9 (C) and QS70 cells (D) with B1QS70 in both light and dark conditions are presented, respectively, with three biological replicates for each condition. DESeq2 software was used to determine different expressed genes, defined as |log_2_FC| > 1 and *p*‐adjusted value < 0.05. GO enrichment analysis of the DEGs of QS9 (E) and QS70 cells (F) under dark treatment is depicted, respectively, with the color and size of dots representing *p* adjust values and the number of enriched genes. Goatools software was used to perform enriched GO terms by Fisher's exact test. The top20 enriched GO terms are shown by corrected *p* adjust value < 0.05. (G) The line chart represents DEGs with the blue line as the average expression in subcluster_1. (H) Volcano plot shows the DEGs of QS9 (+B1QS70) amoebae. Numerals were labeled with green or red letter, representing down‐regulated and up‐regulated gene numbers, respectively. The *y*‐axis represents adjusted *p* values analyzed by DESeq2, and the *x*‐axis shows significant DEGs with |log2FC| > 1. (I) Representative expression of lectin genes (*dscA*, *dscC*, *dscD*, and *dicB*) under light and dark conditions (Data are mean ± SD of 3 independent values, analyzed by DESeq2 with multiple test correction of BH).

To minimize the influence of amoebae morphogenesis, we chose to use amoebae at the same exponential stage (36 h) for RNA sequencing (RNA‐seq) analysis. Amoebae were incubated in light as the control group, while amoebae cells cultured under dark were regarded as the treatment group. The vegetative cells of amoebae QS9 with *P. agricolaris* B1QS70 and the vegetative cells of amoebae QS70 with *P. agricolaris* B1QS70 were collected separately for RNA extraction and transcriptome test after being incubated for 36 h under light and dark conditions. To visualize the differential transcription profiles between the control and treatment groups, the principal component analysis (PCA) was performed. Gene expression patterns under dark and light are clearly separated in PCA analysis. Two principal components (PCs) of QS9 explained 64.56% and 17.01% of the variation, respectively. Meanwhile, the PCs of QS70 explained 73.91% and 8.64% variation, respectively. (Figure 3A,B). This analysis has detected 12,072 genes in total with 11,888 genes annotated and 184 new genes. The proportion of differentially expressed genes (DEGs) was about 6% (721 genes) in amoebae QS9 with symbiont *P. agricolaris* B1QS70, while the proportion of DEGs was about 10% (1205 genes) in amoebae QS70 with symbiont *P. agricolaris* B1QS70. Differentially expressed genes were identified based on at least two‐fold with *p* adjust value below 0.05. These DEGs were then visualized in the heatmap with two main clusters. In amoebae QS9 with *P. agricolaris* B1QS70, 290 genes (40%) were upregulated and 431 genes (60%) were downregulated under dark, resulting in a total of 721 DEGs (Figure 3C). Moreover, in QS70 amoebae with *P. agricolaris* B1QS70, more DEGs (1205 genes) were identified including 435 upregulated genes (36%) and 770 downregulated genes (64%) under dark treatment (Figure 3D). Notably, the number of downregulated genes in both amoebae QS9 and QS70 was at least 1.6 times greater than the number of upregulated genes under dark condition.

The GO enrichment analysis revealed that the differentially expressed genes (DEGs) identified in QS9 and QS70 amoebae with symbiotic *P. agricolaris* B1QS70 play crucial roles in the regulation of various biological processes. The TOP20 enriched GO terms were selected based on the significant DEGs (Figure 3E,F). Interestingly, these DEGs were found to be involved in several biological processes related to symbiotic bacteria, such as intraspecies interaction between organisms, interaction with symbiont, and lectin‐induced modified bacterial internalization. Additionally, DEGs associated with some carbohydrate binding processes were also found to potentially impact the symbiotic status, as the extracellular matrix of bacteria consists of complexed carbohydrates. This indicates that carbohydrate binding proteins from amoebae might have effects on the symbiotic status of *Paraburkholderia* species. It is worth noting that cAMP, acting as a chemotaxis signal of amoebae, plays a key role in cell migration and aggregation. The downregulated acaA (adenylyl cyclase) indicates that amoebae migration or aggregation might be interrupted in the dark. In terms of reproductive processes, genes such as Rho GTPase and Ras GTPase related to cell proliferation were downregulated under dark conditions. Our data demonstrated a significant reduction in the number of germinated spores under dark conditions compared to those in light (Figure S3). This indicates that the downregulated Rho/Ras GTPase could lead to a deficiency in the production of new germinated spores.

Lectin‐induced modified bacteria internalization (LIMBI), as previously reported by Dinh et al., has been found to increase the persistence of symbiotic bacteria in amoebae (Dinh et al. 2018). This suggests that certain lectin genes of amoebae may enhance the formation of bacteria symbiosis and could be regulated by light. Heatmap analysis revealed that 411 genes were downregulated in subcluster_1 under dark (Figure 3G). Notably, several genes associated with decreased symbiont bacteria were found to be downregulated under dark (Figure 3H). For example, lectin genes including *dscA*, *dscC*, *dscD*, and *dicB*, which are involved in the expression of discoidin I, were downregulated under dark (Figure 3I). These findings support the notion that discoidin I could enhance the resistance of *Paraburkholderia* in amoebae cells, as previously reported by Dinh et al. Additionally, other lectin genes like *cupH* could also play a role in regulating *Paraburkholderia* symbiosis in amoebae under light/dark treatment. This indicates that downregulated lectin genes in amoeba QS9 with *P. agricolaris* B1QS70 may reduce symbiosis formation under dark condition. Moreover, when we reestablished the symbiosis between amoeba QS70 and *P. agricolaris* B1QS70, the fluorescence observed in the dark is lower than that in light. Although this difference is not statistically significant, this may also trigger the downregulation of lectin genes in the dark.

### 3.4 Symbiosis of *P. hayleyella* B2QS11‐GFP in QS9, QS11, and QS70 Amoeba Cells Can Be Reduced Under Light Illumination

**FIGURE 4:**
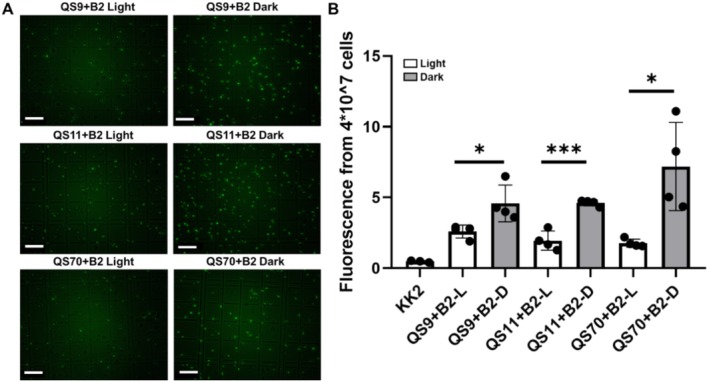
Fluorescence of amoeba cells at the exponential stage (incubated with *Paraburkholderia* B2QS11 for 36 h). (A) The fluorescence images of amoebae QS9, QS11, QS70 with symbiotic B2QS11 were taken under light and dark (scale bar = 40 μm). (B) The fluorescence of a certain amount of amoebae cells (QS9, QS11, QS70, normalized to 4 × 10^7^) with symbiotic B2QS11 was detected under dark and light. Statistical significance was determined using Student's *t*‐test. Data are mean ± SD of 3–4 independent experiments (*p < 0.05, ***p < 0.001).

*P. hayleyella* B2QS11 exhibits a higher level of infectivity towards amoebae compared to *P. agricolaris* B1QS70. Unlike the symbiosis formation observed in amoebae infected with B1QS70, the growth rate of B2QS11 is notably enhanced under dark conditions. Consequently, all the amoebae clones QS9, QS11, and QS70 were strongly infected by *P. hayleyella* B2QS11 at the exponential stage after 36 h. Fluorescence imaging clearly illustrates a higher fluorescence intensity in amoebae cells under dark conditions compared to light conditions (Figure 4A). Moreover, fluorescence measurements were conducted using an equal number of vegetative amoebae cells (4*10^7^), which revealed that the fluorescence intensity under dark is at least 2‐fold higher than that in the light (Figure 4B). These data showed that the fluorescence of amoebae cells is significantly higher in constant darkness compared to constant light.

To compare the dynamics between new exposure to symbionts (*P. hayleyella* B2QS11 in amoeba QS9) and an established symbiosis (*P. hayleyella* B2QS11 in amoeba QS11), we investigated type II symbiosis following acclimation over two social cycles. We analyzed the fluorescence of amoeba QS9 with symbiotic B2QS11‐GFP across the two social cycles and found that the fluorescence of symbiont B2QS11 in QS9 was enhanced during dark incubation compared to light treatment in the first and second social cycles (Figure S11). Similarly, we assessed the fluorescence of amoeba QS11 with symbiotic B2QS11‐GFP across the two social cycles. While we observed that the fluorescence of symbiont B2QS11 in QS11 was also enhanced under dark incubation compared to light treatment in the first and second social cycles, this enhancement was not as strong as that observed in QS9 with B2QS11 (Figure S12). However, throughout both dynamic symbiosis tests, we noted a decrease in the number of fruiting bodies and a reduction in spores in the second social cycle, which is difficult to continuously culture in the third social cycle under dark conditions. We suggested that *B. hayleyella* B2QS11 is more detrimental to *D. discoideum* hosts, so the newly established symbiosis of B2QS11 in QS9 relies primarily on the amount of *B. hayleyella* B2QS11 in the incubation culture. Therefore, the type II symbiosis appears to be mainly dependent on the appropriate abundance of *B. hayleyella* B2QS11. We hypothesize that the symbiont's dose‐dependent pattern may trigger type II symbiosis in a natural soil environment. Future experiments could explore the precise amount of symbiont required to initiate this symbiosis. At the 7‐day fruiting body stage, the images displayed a stronger fluorescence similar to that observed during the single‐cell stage when the amoebae with B2QS11 were incubated in the dark (Figure S13B). When examining spores taken from fruiting bodies, it is reasonable to observe that the fluorescence of QS9, QS11, and QS70 spores would be higher in the dark compared to in the light (Figure S14). The fluorescence of amoebae with *P. hayleyella* B2QS11 in constant dark was significantly higher than in constant light (Figure S13A). Interestingly, we observed that the fluorescence of QS9, QS11, and QS70 with B2QS11 is still significantly reduced when the incubation time was reduced from constant dark to 2 days of dark incubation after 5 days of light exposure (L5D2) (Figure S13A). These findings suggest that the final symbiosis in the life cycle of host amoebae may be determined by the dynamic growth rate of *P. hayleyella* B2QS11.

### 3.5 Transcriptome Profiles of QS9 and QS11 With *P. hayleyella* B2QS11 at the Exponential Stage Under Light and Dark

**FIGURE 5:**
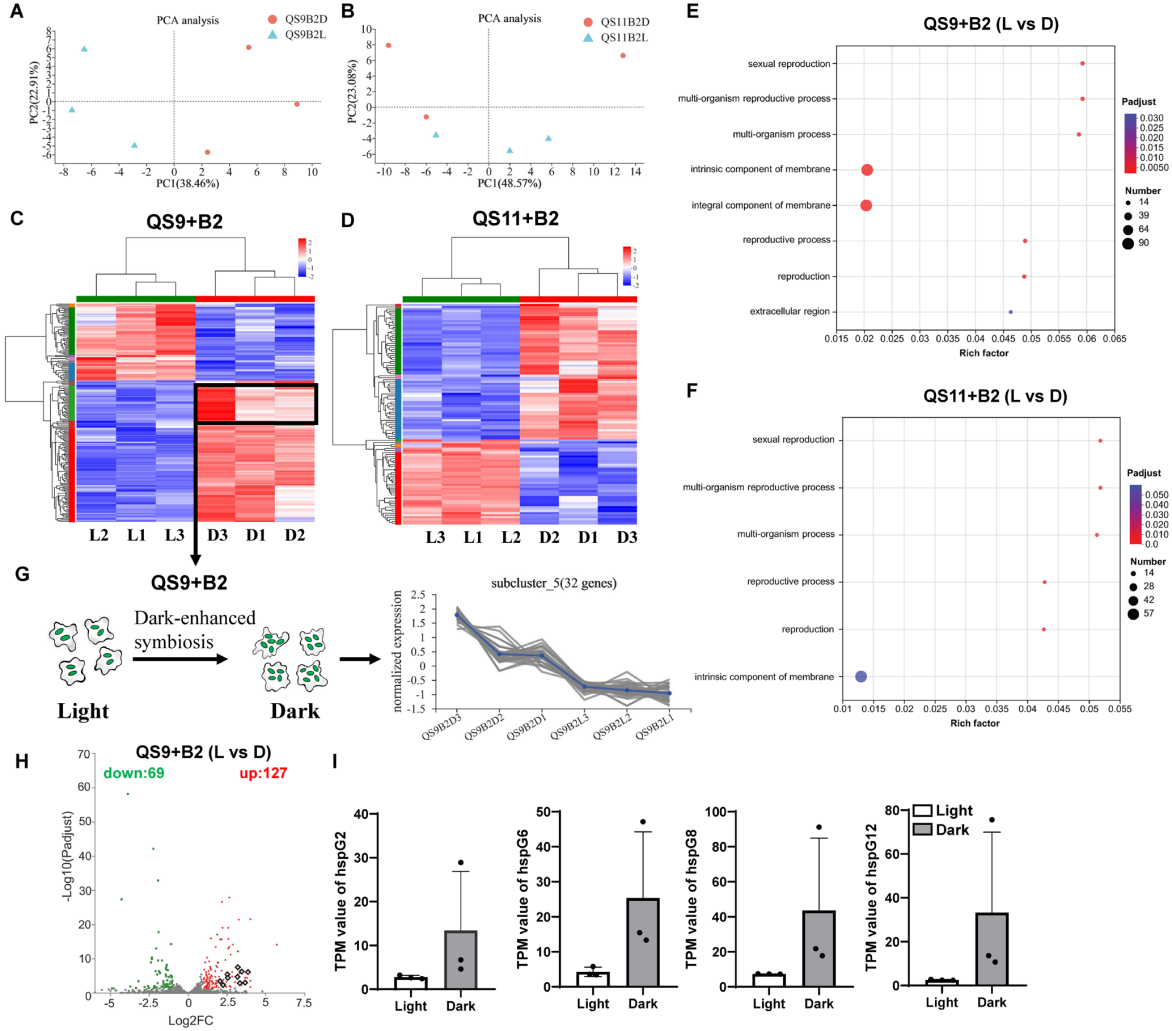
Transcriptome profiles of QS9 and QS11 amoebae with symbiotic B2QS11. Three biological replications were performed under light as the control and dark as the treatment, respectively. PCA was performed on RNA‐seq datasets from QS9 (A) and QS11 cells (B) with symbiotic B2QS11 under light and dark conditions, respectively. Heatmaps displaying differentially expressed genes (DEGs) from QS9 (C) and QS11 cells (D) with B2QS11 in light and dark conditions are presented, with three biological replicates for each condition. DESeq2 software was used to determine different expressed genes, defined as |log_2_FC| > 1 and *p*‐adjusted value < 0.05. GO enrichment analyses of the DEGs of QS9 (E) and QS11 cells (F) under dark treatment are illustrated, respectively, with the color and size of dots indicating *p* adjust values and the number of enriched genes. Goatools software was used to perform enriched GO terms by Fisher's exact test. The enriched GO terms are shown by corrected *p* adjust value < 0.05. (G) The line chart represents DEGs with the blue line as the average expression in subcluster_5. (H) Volcano plot shows the DEGs of QS9 (+B2QS11) amoebae. Numerals were labeled with green or red letter, representing down‐regulated genes and up‐regulated genes, respectively. The *y*‐axis represents adjusted *p* values analyzed by DESeq2, and the *x*‐axis shows significant DEGs with |log_2_FC| > 1. (I) Expression of representative heat shock genes (*hspG2*, *hspG6*, *hspG8*, and *hspG12*) under light and dark conditions (data are mean ± SD of 3 independent values, analyzed by DESeq2 with multiple test correction of BH).

Due to the distinct symbiosis and infection of *P. hayleyella* B2QS11 in amoebae, it is evident that we will observe different transcriptome profiles compared to *P. agricolaris* B1QS70 symbiosed amoebae. We utilized vegetative amoebae at the unicellular stage for RNA‐seq. The control group was exposed to light, while the treatment group was kept in the dark. Here we mixed individual QS9 and QS11 amoebae cells with *P. hayleyella* B2QS11 for 36 h under light and dark conditions, and RNA extraction was conducted in the vegetative cells. To compare the transcription profiles between light and dark treatments, principal component analysis was performed. The two principal components (PCs) of QS9 explained 38.46% and 22.91% of the variation, respectively. Meanwhile, the PCs of QS11 explained 48.57% and 23.08% variation, respectively (Figure 5A,B). This analysis has detected 12,044 genes in total with 11,863 genes annotated and 181 new genes. The proportion of differentially expressed genes (DEGs) was about 1.6% (196 genes) in amoeba QS9 with symbiont *P. hayleyella* B2QS11, while the proportion of DEGs was about 0.9% (109 genes) in amoeba QS11 with symbiont *P. hayleyella* B2QS11. Differently expressed genes were adjusted to at least 2‐fold change with *p*‐adjust value below 0.05. The DEGs were constructed and shown in the heatmap with two main clusters. In QS9 amoebae with *P. hayleyella* B2QS11, 127 genes (65%) were upregulated and 69 genes (35%) were downregulated under dark, resulting in a total of 196 DEGs (Figure 5C). Moreover, in QS11 amoebae with *P. hayleyella* B2QS11, fewer DEGs (109 genes) were identified, including 67 upregulated genes (61%) and 42 downregulated genes (39%) under dark treatment (Figure 5D). It is clear to observe the number of upregulated genes in both QS9 and QS11 is at least 1.6 times greater than the number of downregulated genes under dark condition. This is in contrast to the symbiosed *P. agricolaris* B1QS70 in amoebae. Therefore, we focused on some upregulated DEGs.

GO enrichment analysis showed that some key genes, such as EGF‐like domain‐containing protein coding genes, were upregulated and played key roles in regulating biological processes involved in intrinsic component of membrane, sexual reproduction, and reproduction process etc. (Figure 5E,F). It was expected that lectin genes might be influenced similarly to *P. agricolaris* B1QS70 symbiosis, but those typical lectin genes were not significantly expressed under dark. We observed that 32 genes were upregulated under dark in subcluster_5 (Figure 5G). Some heat shock genes related to the pathogen infection were upregulated and caused immune responses under dark treatment for *P. hayleyella* B2QS11 symbiosed amoebae QS9 (Figure 5H). For example, heat shock genes including *hspG2*, *hspG6*, *hspG8*, *hspG12* were upregulated under dark (Figure 5I). Heat shock proteins typically respond to immune responses in animal systems. Therefore, it is reasonable to observe no significant gene expression of those heat shock genes in amoebae QS11 because it is the native host of *P. hayleyella* B2QS11 with a stable symbiosis status. This may result in fewer or no immune responses compared to *P. hayleyella* B2QS11 symbiosis in the non‐native host QS9. The combination of amoebae and symbiotic *Paraburkholderia* indicates that the two light‐regulated symbiosis types between *P. agricolaris* B1QS70 and *P. hayleyella* B2QS11 can elicit different responses in amoebae.

**FIGURE 6:**
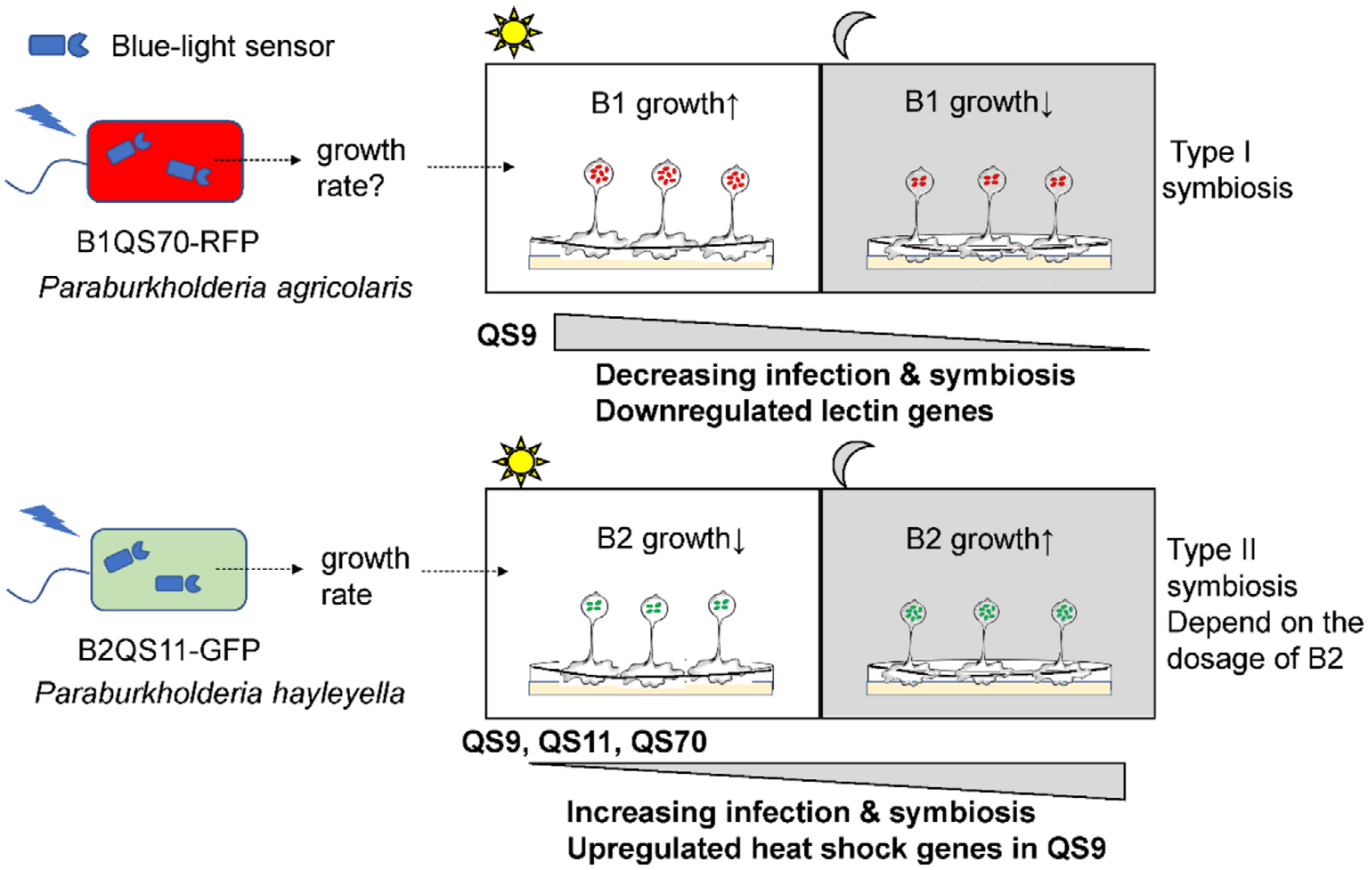
The model of two types of light‐regulated symbiosis for the interaction between amoebae and *Paraburkholderia*. Type I symbiosis indicates that light can enhance the symbiotic relationship between B1QS70 and QS9. Type II symbiosis suggests that the symbiotic relationship between B2QS11 and the three amoebae clones (QS9, QS11 and QS70) is increased in the dark, with the growth rate of B2QS11 playing a significant role in this process.

In conclusion, we found that the symbiosis status of two distinct *Paraburkholderia* species, B1QS70 and B2QS11, is different due to their infection efficiency. Based on our findings, we proposed two light‐regulated symbiosis types, which are influenced by the level of infection (Figure 6). Light may play key roles in regulating the symbiosis because of light sensors in *Paraburkholderia* species. In type I symbiosis, light can only enhance the symbiotic relationship between *P. agricolaris* B1QS70 and amoeba QS9, but it does not have the same effect on the symbiosis between amoebae QS11 and QS70 with their native symbiont in the natural environment. This indicates that light may improve the symbiosis or adaptation between amoeba QS9 and *P. agricolaris* B1QS70 in the ecological niche. The stability of symbiosis may have been gradually established with an acclimation period in light, as indicated by the presence of amoebae QS11 and QS70 with their native endosymbiotic bacterium without the effects of light regulation in most cases. However, in type II symbiosis involving *P. hayleyella* B2QS11, we observed that light strongly inhibits the symbiotic relationship and leads to less infection when *P. hayleyella* B2QS11 is mixed with amoebae cells. This inhibition is likely due to the light‐regulated growth conditions of *P. hayleyella* B2QS11 and its high infection efficiency. Through sequence alignment, some conserved blue light photoreceptors were identified from 18 different *Paraburkholderia* species (Figure S15), which belong to the family of cryptochrome/DNA photolyase. This may indicate that the growth of *P. hayleyella* B2QS11 may possibly be inhibited by blue light.

## 4 Discussion

As unicellular or multicellular protozoa, amoebae are widely distributed in various environments and primarily feed on a wide range of bacteria. In a chemotaxis assay, it was observed that *D. discoideum* prefers Gram‐negative bacteria with a higher hatching rate and more spores compared to Gram‐positive bacteria (Rashidi and Ostrowski 2019; Shu et al. 2021). Some other amoeba‐resistant bacteria (ARBs) have evolved to survive within amoebae cells through specific mechanisms (Tosetti et al. 2014). Various environmental cues have effects on the development and symbiotic relationship between amoebae and bacteria. For instance, nano‐ and microplastics present in soil ecosystems have been found to affect the development of amoebae (Zhang et al. 2022). The intracellular bacteria can be eliminated by disinfection techniques employed in drinking water systems (He et al. 2021). However, the long‐term impact of environmental factors on amoebae‐bacteria symbiosis remains poorly understood. Our data analysis provides evidence that light stimulation is a crucial factor in dynamically altering the interactions between amoebae and bacteria. In the non‐natural symbiotic amoebae QS9, the symbiosis of *P. agricolaris* B1QS70 is dramatically increasing under light incubation compared to dark incubation. Conversely, the symbiosis of *P. agricolaris* B1QS70 remains stable under dark and light in the natural hosts QS11 and QS70. This suggests that light may play a key role in establishing a stable symbiotic relationship over long periods of evolution. The stable symbiosis between amoebae and symbiotic bacteria is more likely to be selected through prolonged interactions influenced by light stimulation.

Previous study has demonstrated that two different clades of *Paraburkholderia* species emerged by 16S rRNA gene phylogeny. This suggests the possibility of two independent origins of symbiosis between *Paraburkholderia* and *D. discoideum* (DiSalvo et al. 2015). Therefore, we chose to use *P. agricolaris* (B1QS70) and *P. hayleyella* (B2QS11) as the symbiotic bacteria to be incubated with amoebae QS9, QS11, and QS70. We hypothesize that these two distinct *Paraburkholderia* species may exhibit different types of symbiosis due to their significantly different genome sizes. Specifically, *P. hayleyella*, which has a genome size approximately half that of *P. agricolaris*, demonstrates a stronger infection rate towards the native amoebae hosts. This indicates that *P. hayleyella* may rely more heavily on the amoebae hosts compared to *P. agricolaris*. Our data further revealed that the fluorescence of *P. hayleyella* B2QS11 inside amoebae increased in darkness, corresponding to the growth rate of *P. hayleyella* B2QS11 itself.

In type I symbiosis, we propose that light plays a crucial role in enhancing the interaction between amoebae QS9 and *P. agricolaris* B1QS70. In three amoebae QS9, QS11, and QS70 with or without symbiotic *Paraburkholderia* species, we observed that light significantly increased the production of new germinated spores by approximately 3–5 times compared to the spore number in dark incubation (Figure S3). For non‐symbiotic amoebae QS9, the *P. agricolaris* B1QS70 was increasing under light illumination, corresponding to the enhanced growth of amoebae spores QS9. The stability of *P. agricolaris* B1QS70 was maintained in amoebae QS11 and QS70, suggesting a possible long‐term evolutionary adaptation to their natural symbiotic *Paraburkholderia* species. After comparing the dynamics of symbiosis following acclimation over three social cycles, we propose that the light‐enhanced fluorescence of symbiont B1QS70 in QS70 may also be observed in the third social cycle (Figure S5). This suggests that light‐induced type I symbiosis could also happen in specific social cycles of QS70 with symbiont B1QS70 during the acclimation period for the establishment of stable symbiosis.

Previous studies have shown that the interactions between *D. discoideum* and bacteria are regulated by specific signals. For example, defensive symbionts of amoeba farmers can protect their crops (Brock et al. 2013). The inedible symbiotic bacteria can be transformed into a food strain by generating a knockout mutant of the global activator (gacA) (Stallforth et al. 2013). Furthermore, it has been suggested that the microbiome homeostasis of social amoebae may be influenced by endogenous or environmental lectins (Dinh et al. 2018). In our transcriptome data, we observed that lectin genes were downregulated under dark conditions when the fluorescence of symbiotic *P. agricolaris* B1QS70‐RFP was reduced in amoebae. Therefore, one potential mechanism underlying the interaction between non‐symbiotic amoeba QS9 and *P. agricolaris* B1QS70 could involve the upregulated lectin genes in the host under light illumination.

In the type II symbiosis, we observed an increase in fluorescence of *P. hayleyella* B2QS11 in amoebae under dark conditions when we combined it with each individual amoeba QS9, QS11, and QS70. We also performed dynamic symbiosis tests comparing new exposure to symbiosis (B2QS11 in amoeba QS9) (Figure S11) and an established symbiosis (B2QS11 in amoeba QS11) (Figure S12) after acclimation over two social cycles. Our data indicate that type II symbiosis is primarily dependent on the appropriate amount of *B. hayleyella* B2QS11. We propose that the higher abundance of symbionts in the culture plate would be detrimental to the amoeba host across multiple social cycles, which differs from the symbiont dosage found in amoebae isolated from the natural soil environment where symbiosis is sustainably established. We suggested that the dose‐dependent pattern of symbiont B2QS11 may trigger type II symbiosis in natural soil environments. Exploring the precise amount of symbiont required to initiate symbiosis in culture plates would be an interesting focus for future experimental investigation.

It appears that *P. hayleyella* B2QS11 isolates may have a stronger infective effect on native non‐symbiotic amoebae hosts compared to evolved symbiotic hosts (DiSalvo et al. 2015). This could make amoebae cells generate different immune responses to various bacteria, especially symbiotic *Paraburkholderia* species. *D. discoideum* cells exhibit immune responses that are evolutionarily conserved and are similar to phagocytic cells in humans (Dunn et al. 2018). Given that the interactions between amoebae and bacteria have existed for a long time, it is possible that some bacteria have evolved mechanisms to trigger immune responses in amoebae. Heat shock proteins are typically expressed when organisms respond to certain stimuli, including pathogenic bacteria (Bolhassani and Agi 2019; Kaufmann 1990). We hypothesized that *P. hayleyella* B2QS11 could induce stronger immune responses in non‐symbiotic amoeba QS9 compared to its native symbiotic amoeba QS11. Transcriptome analysis revealed that some heat shock genes (HKGs) were upregulated in amoebae QS9 with *P. hayleyella* B2QS11, but not in QS11 with B2QS11 under dark. Therefore, it is suggested that the interaction between amoeba QS9 and *P. hayleyella* B2QS11 may rely on the heat shock proteins during a strong infection.

Photoreceptors play key roles in regulating the physiological conditions of algae, bacteria, fungi, and plants response to dark and light environments. Some typical photoreceptors, including blue light‐gated cryptochromes and red light‐gated phytochromes, are important in controlling various cellular processes (Shcherbakova et al. 2015). Some blue light sensors belonging to cryptochromes/photolyase family members were identified in the protein database of *P. agricolaris* and *P. hayleyella*, while other photoreceptors stimulated by different light wavelengths were not found in the database. Therefore, the blue light could influence the growth conditions of *Paraburkholderia* species. We observed that blue light can reduce the growth rate of *P. hayleyella* B2QS11, but it does not affect the growth of *P. agricolaris* B1QS70. This suggests that the interaction between amoebae and *P. hayleyella* B2QS11 is diminished under light illumination due to the decreased growth of B2QS11. Different strains of *P. hayleyella* may exhibit distinct growth rates under light and dark conditions, possibly due to different photoreceptors. Future investigations would be more interested in establishing symbiosis among a broader range of fluorescently tagged *P. hayleyella* strains and amoebae clones under both light and dark conditions. On the other hand, the non‐symbiotic amoebae QS9‐B1QS70 interaction primarily relies on the growth of amoebae under light incubation, while the two symbiotic strains QS11 and QS70 can sometimes maintain stability with *P. agricolaris* B1QS70 in nature, with the acclimation period for the establishment of stable symbiosis.

*D. discoideum* is an ideal model organism for studying symbiosis, which allows us to better understand the ecology and evolution of the relationship between host and bacteria (Haselkorn et al. 2019). This well‐established symbiotic system enables us to understand how the growth of amoebae influences the symbiotic *P. agricolaris* or vice versa. Our data reveal that light can have effects on the dynamically symbiotic relationships between amoebae and *Paraburkholderia* species. Various types of symbiosis of amoeba‐bacteria interaction were detected under different light responses. Light–dark cycles have been shown to regulate circadian rhythms and physiological conditions in almost all kingdoms of life (Bumgarner and Nelson 2021). This observation suggests that the long‐term coevolution of host‐bacteria interactions may be influenced by light or dark conditions. Constant light illumination has been found to promote the formation of the long‐established symbiosis between non‐symbiotic amoebae QS9 and *P. agricolaris* B1QS70, similar to the symbiosis observed in amoebae QS70 and B1QS70. However, constant darkness promotes the symbiosis between *P. hayleyella* B2QS11 and three tested amoebae due to its stimulatory effect on growth. Therefore, it is crucial to consider light conditions as key factors contributing to the long‐time evolutionary status of both protist‐bacteria interactions and higher eukaryotes such as plants and animals with symbiotic microorganisms. Investigating the mechanisms underlying light‐induced or dark‐induced host‐bacteria interactions may provide valuable insights into key ecological questions.

Overall, the establishment of symbiotic relationships between host and bacteria may be influenced by the circadian rhythm, suggesting that light could potentially serve as a crucial regulator of host‐bacteria interactions. To better understand how light exposure can impact symbiosis, we proposed two light‐induced symbiosis types between social amoebae and *Paraburkholderia* species. We suggested that the type I symbiosis demonstrates that light illumination can significantly increase the amount of *P. agricolaris* B1QS70 in native amoebae QS9, likely due to the host amoebae metabolites such as lectins. Conversely, the type II symbiosis reveals that light incubation could reduce the amount of *P. hayleyella* B2QS11 in three individual amoebae clones (QS9, QS11, or QS70), probably because of reduced amounts of highly infectious B2QS11 under light conditions. This study indicates that the establishment of host–microbe symbiosis may rely on light exposure and their own individual host metabolites or bacterial infections.

## Author Contributions

**Yuehui Tian:** conceptualization (equal), data curation (equal), formal analysis (equal), investigation (equal), visualization (equal), writing – original draft (equal). **Lin Zhang:** data curation (equal), formal analysis (equal), visualization (equal), writing – original draft (equal). **Zihe Wang:** data curation (equal), investigation (equal), methodology (equal), writing – original draft (equal). **Zhili He:** formal analysis (equal), investigation (equal), writing – review and editing (equal). **Longfei Shu:** conceptualization (equal), data curation (equal), funding acquisition (equal), investigation (equal), project administration (equal), supervision (equal), writing – original draft (equal), writing – review and editing (equal).

## Conflicts of Interest

The authors declare no conflicts of interest.

## Supporting information


Figures S1–S15.

Data S1.

File S1.

File S2.

## Data Availability

All data and figures produced or analyzed during this research are contained within this article and its supplementary files. The raw sequence data of transcriptomic analysis reported in this paper have been deposited in the Genome Sequence Archive (Chen et al. 2021) in the National Genomics Data Center (Members and Partners 2024), the China National Center for Bioinformation/Beijing Institute of Genomics, the Chinese Academy of Sciences (GSA: CRA022670) that are publicly accessible at https://ngdc.cncb.ac.cn/gsa.
