# Supplementary material for: Light Affects Host‐Symbiont Dynamics in the Non‐Photosynthetic Social Amoeba Symbiosis
Source: Ecol Evol. 2025 Apr 18;15(4):e71320. doi: 10.1002/ece3.71320 (PMC12008035; doi:10.1002/ece3.71320)
Supplement: Supplementary file 1 — Figures S1–S15. [file ECE3-15-e71320-s004.docx]

Supplemental materials for

**Light affects** **host-symbiont dynamics in the non-photosynthetic** **social amoeba symbiosis**

**Supplementary Figures**


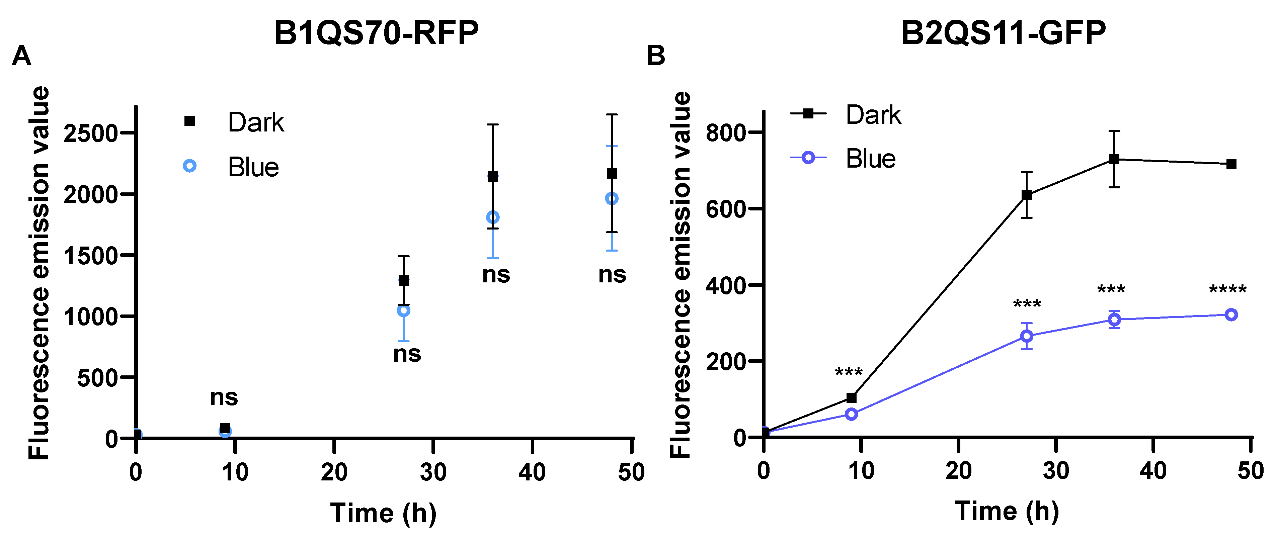


**Figure S1.** Fluorescence measurement of B1QS70-RFP, B2QS11-GFP. For (A) and (B), the incubation time points under dark and blue light at 21 ℃ are operated at 9 h, 27 h, 36 h and 48 h (Multiple t-tests between identical groups in each time point were performed. Three biological replications were operated in each time point, n = 3; all error bars represent the SD. ns indicates no significance; ***, P < 0.001; ****, P < 0.0001).


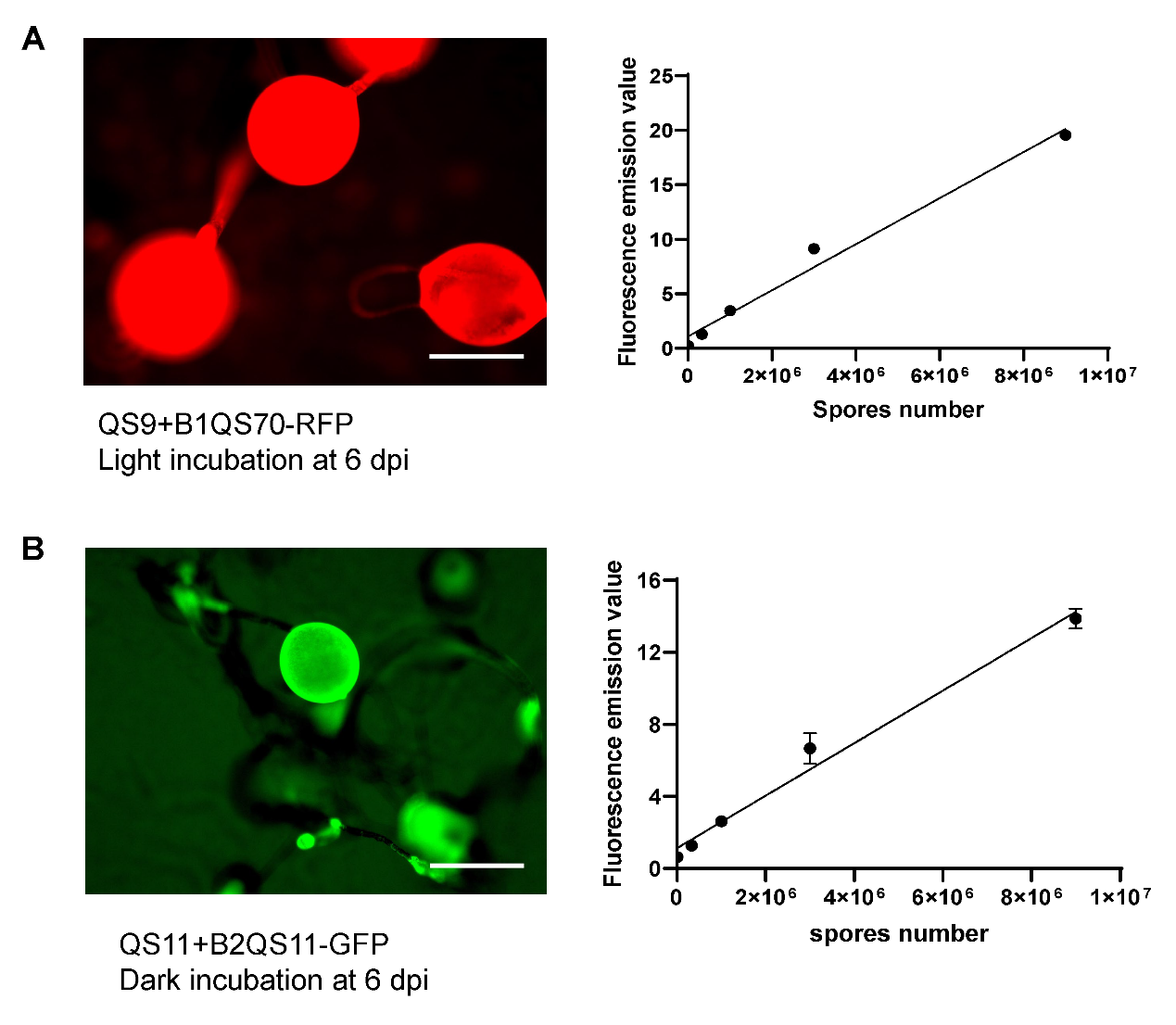


**Figure S2**. Fluorescence measurement of amoebae spores taken from fruiting body after 6 days post incubation (dpi). (A) amoebae QS9 with B1QS70-RFP was incubated in light for 6 days (scale bar=300 μm). (B) QS11 with B2QS11-GFP was incubated under dark for 6 days. For both (A) and (B), the spore numbers with 3.3*10^5^, 1*10^6^, 3*10^6^ and 9*10^6^ were used for fluorescence measurement respectively (Three technical replications were performed, n = 3; all error bars represent the SD).


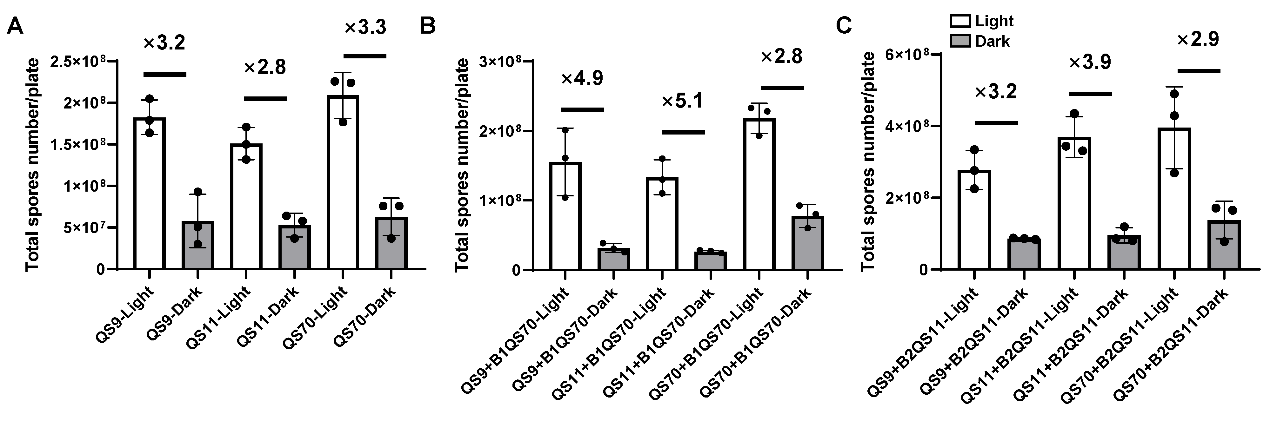


**Figure S3.** Total amoebae spore numbers are enhanced by light. (A) detection of total spore numbers (QS9, QS11, and QS70) without symbiotic bacteria in individual plates under light and dark. (B) total spore numbers (QS9, QS11, and QS70) with symbiotic B1QS70 under dark and light. (C) total spore numbers (QS9, QS11, and QS70) with symbiotic B2QS11 under dark and light. For (A)-(C), individual data point represents each biological replicate (Three biological replications were performed, n = 3; all error bars represent the SD).


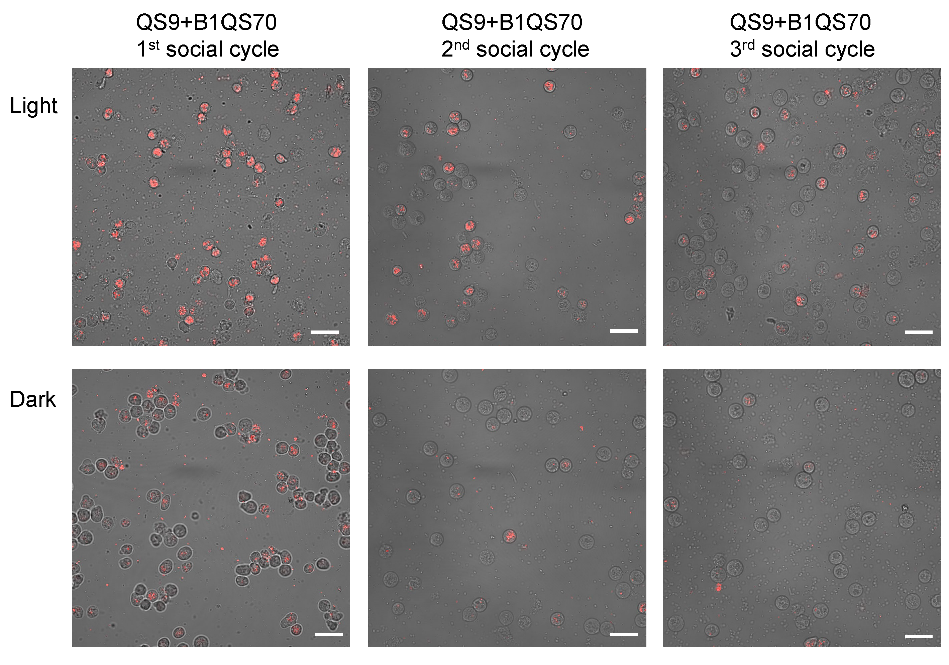


**Figure S4**. Fluorescence of amoeba QS9 cells at the exponential stage (incubated with *Paraburkholderia* B1QS70 for 36 h) was measured under light and dark treatments across three social cycles. Three individual experiments were showed similar trends in fluorescence changes (scale bar=20 μm).


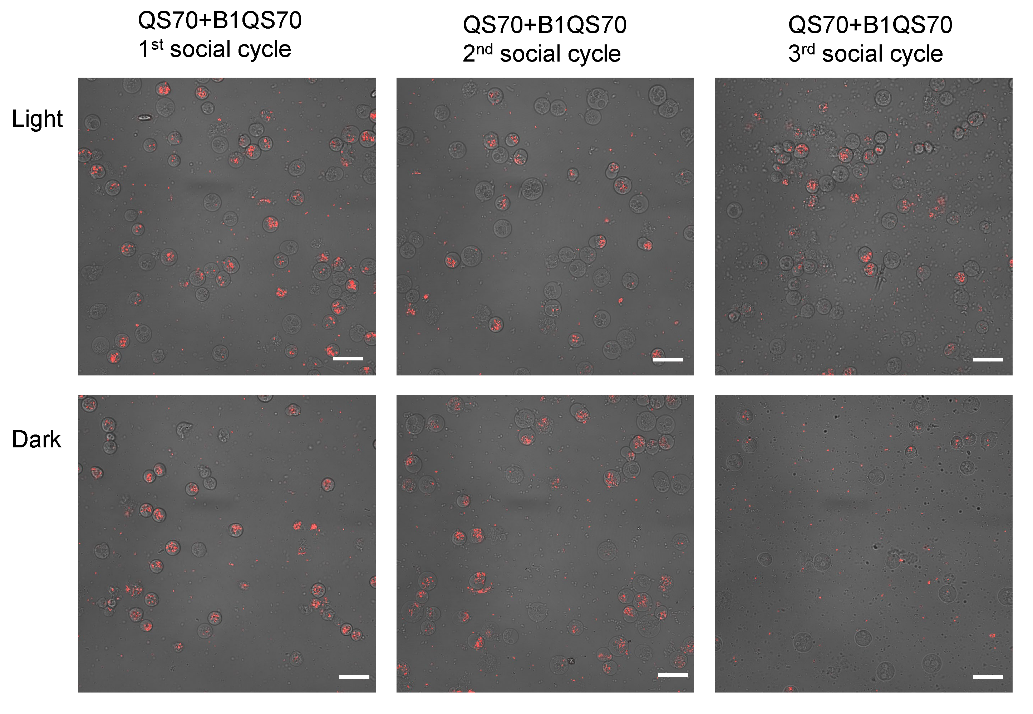


**Figure S5**. Fluorescence of amoeba QS70 cells at the exponential stage (incubated with *Paraburkholderia* B1QS70 for 36 h) was measured under light and dark treatments over three social cycles. Three individual experiments were investigated with similar trends in fluorescence changes (scale bar=20 μm).


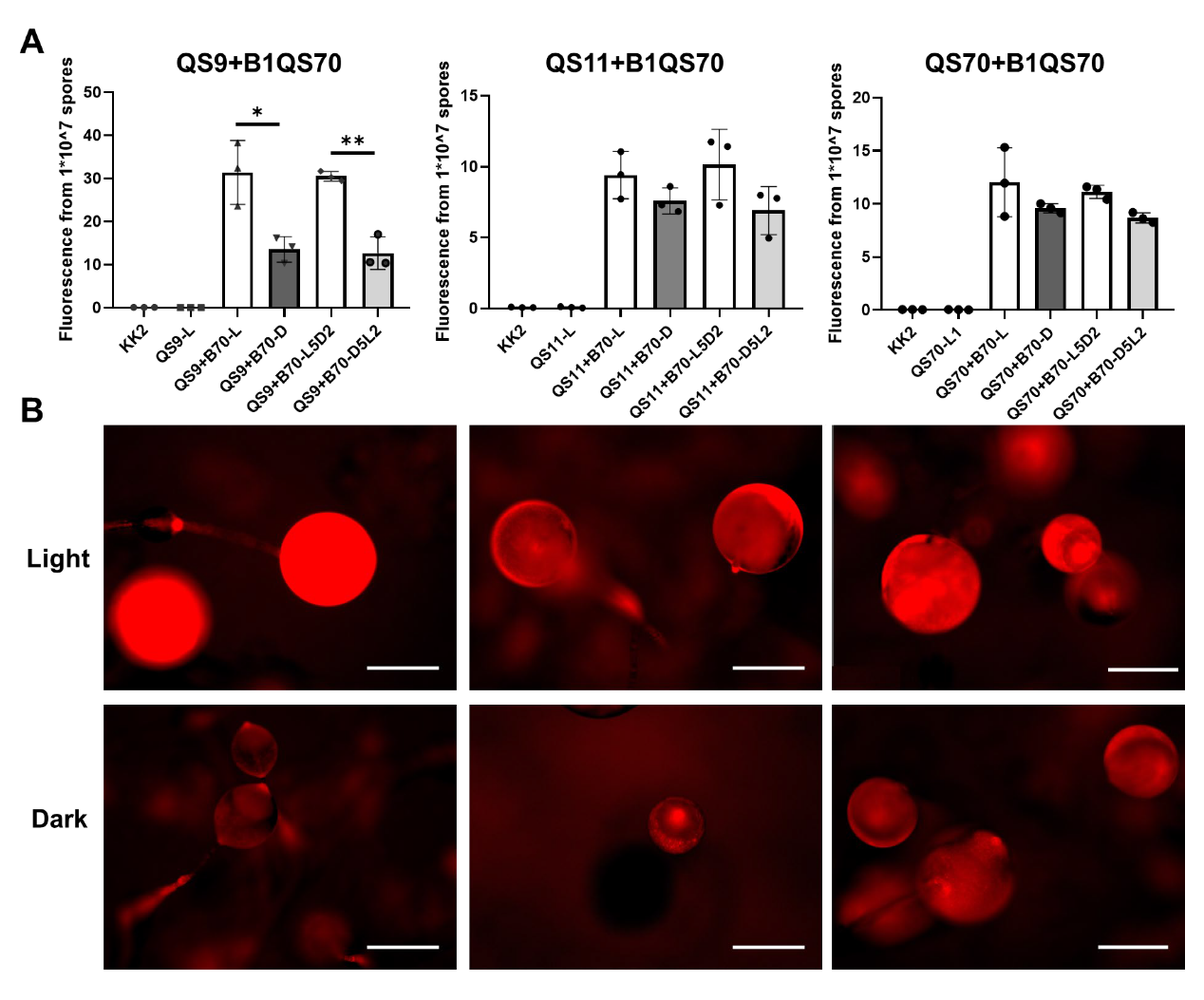


**Figure S6.** Fluorescence of amoeba spores in the fruiting body (incubated with *Paraburkholderia* B1QS70 for 7 days or as indicated). (A) Fluorescence measurement in certain amount of new germinated spores (QS9, QS11, QS70, 1×10^7^) with symbiotic B1QS70 under dark and light (Three biological replications were performed, n = 3; all error bars represent the SD). Asterisks indicate significance (*, P < 0.05; **, P < 0.01) according to the one-way ANOVA Tukey’s multiple-comparison test. KK2, QS9, QS11, and QS70 without symbiotic bacteria represent the blank and negative controls. L5D2 indicates 5 days light incubation and 2 days dark condition. D5L2 indicates 5 days dark incubation and 2 days light illumination. (B) Fluorescence images of QS9, QS11, and QS70 fruiting body with symbiotic B1QS70 were taken under light and dark (scale bar=300 μm).


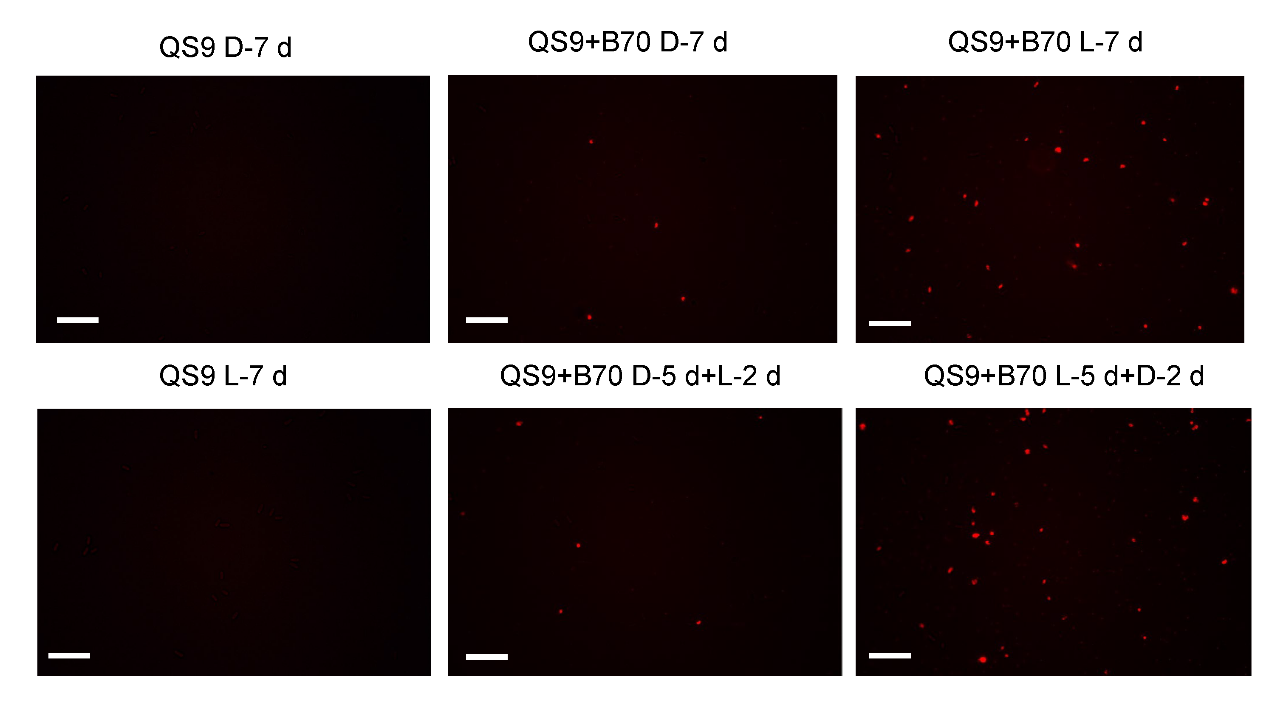


**Figure S7**. Intracellular B1QS70-RFP increased in QS9 amoeba spores under light illumination at 7 d. QS9 without symbiotic B1QS70 as the negative control. The fluorescence of QS9+B1QS70 (5 days dark and 2 days light) was similar with constant dark condition for 7 days. The fluorescence of QS9+B1QS70 (5 days light and 2 days dark) was similar with constant light illumination for 7 days (scale bar=40 μm).


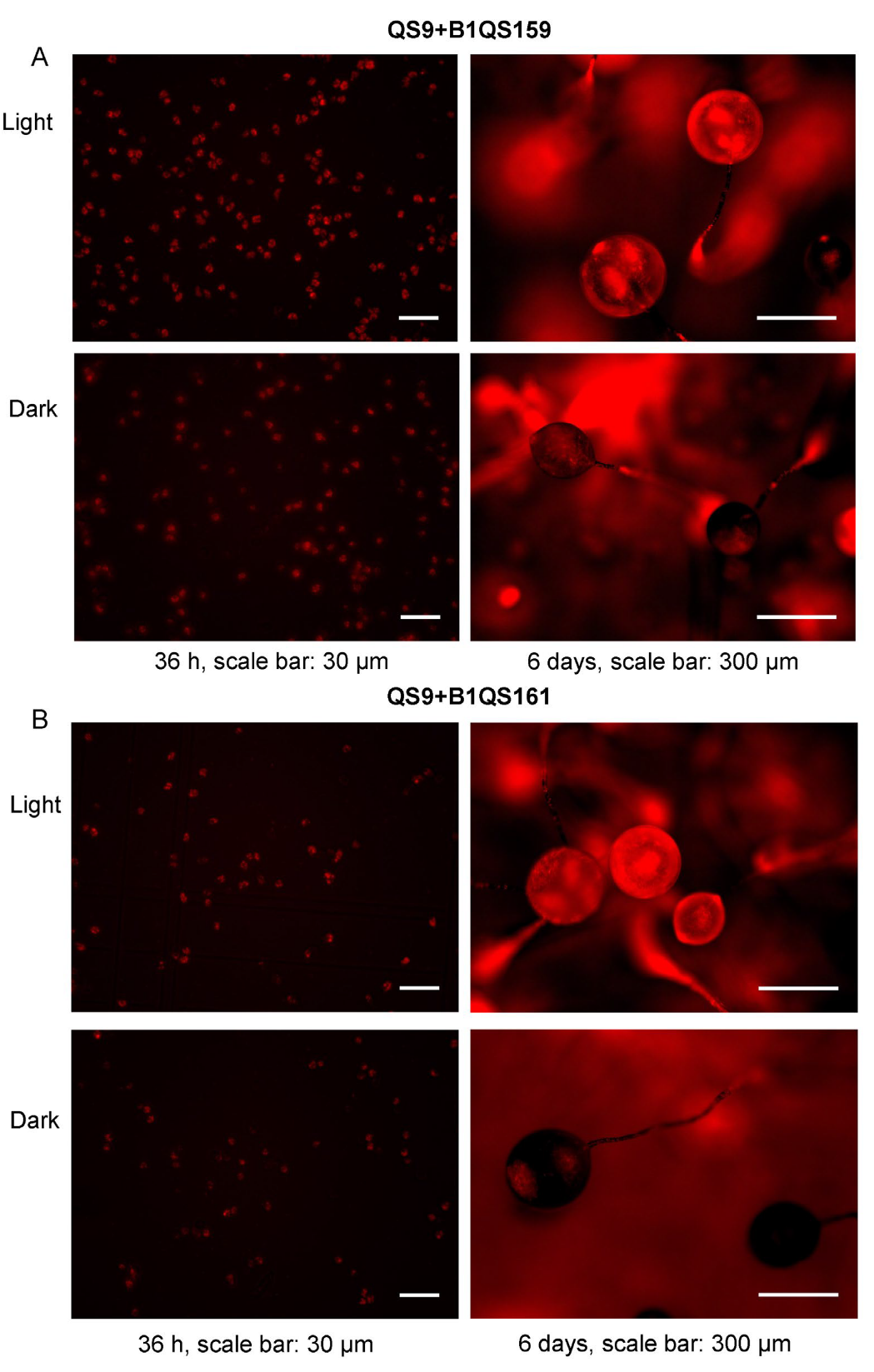


**Figure S8**. The fluorescence of two symbiotic bacteria, B1QS159-RFP (A) and B1QS161-RFP (B), was evaluated in amoeba QS9 vegetative cells and fruiting bodies under both light illumination and dark incubation treatments. Three biological replications were performed in each pairing, producing similar fluorescence detection results.


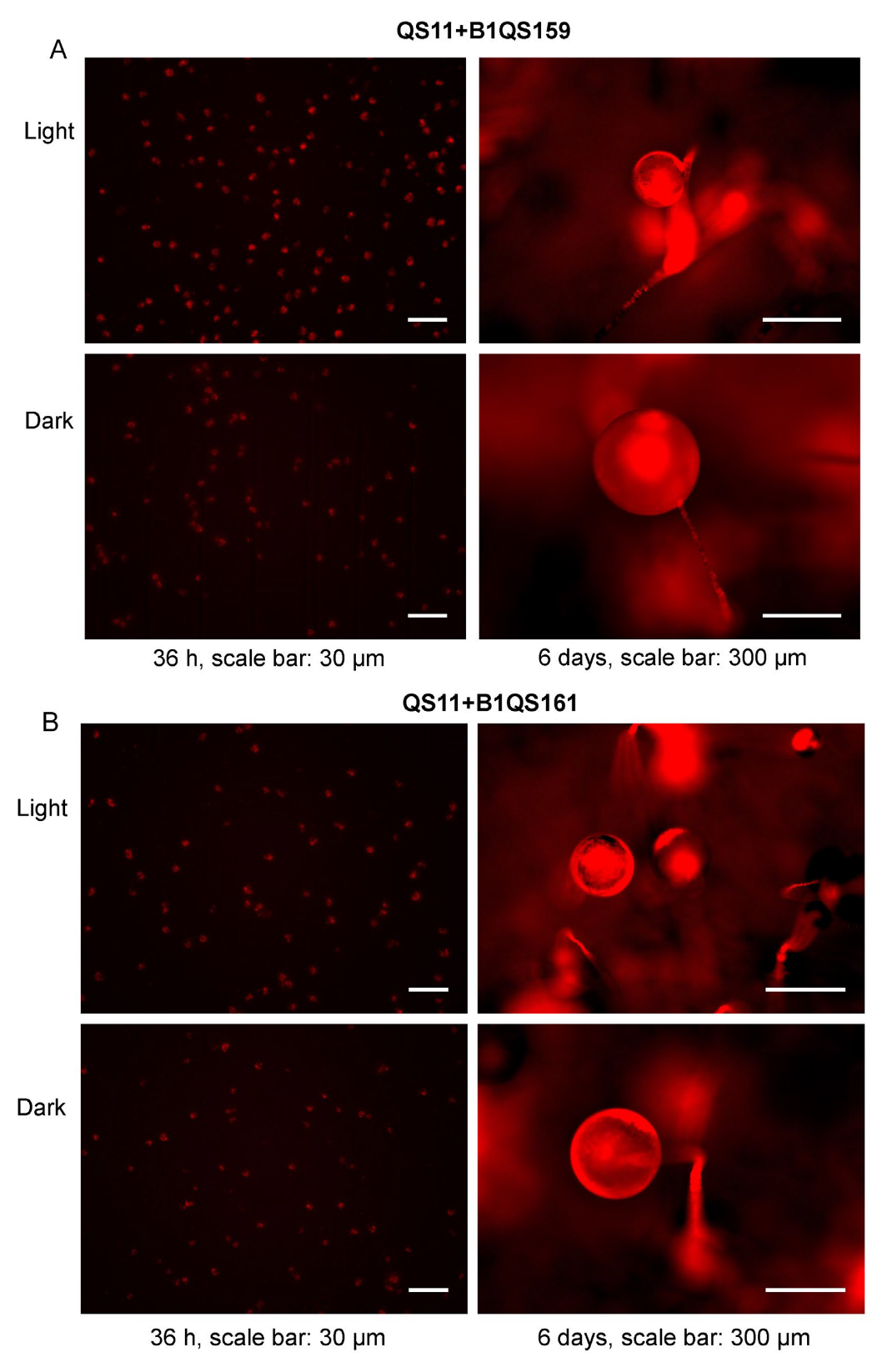


**Figure S9**. The fluorescence of two symbiotic bacteria B1QS159 (A) and B1QS161 (B) was assessed in amoeba QS11 vegetative and fruiting bodies under both light and dark treatments. Three biological replications were performed in each pairing, producing consistent fluorescence detection.


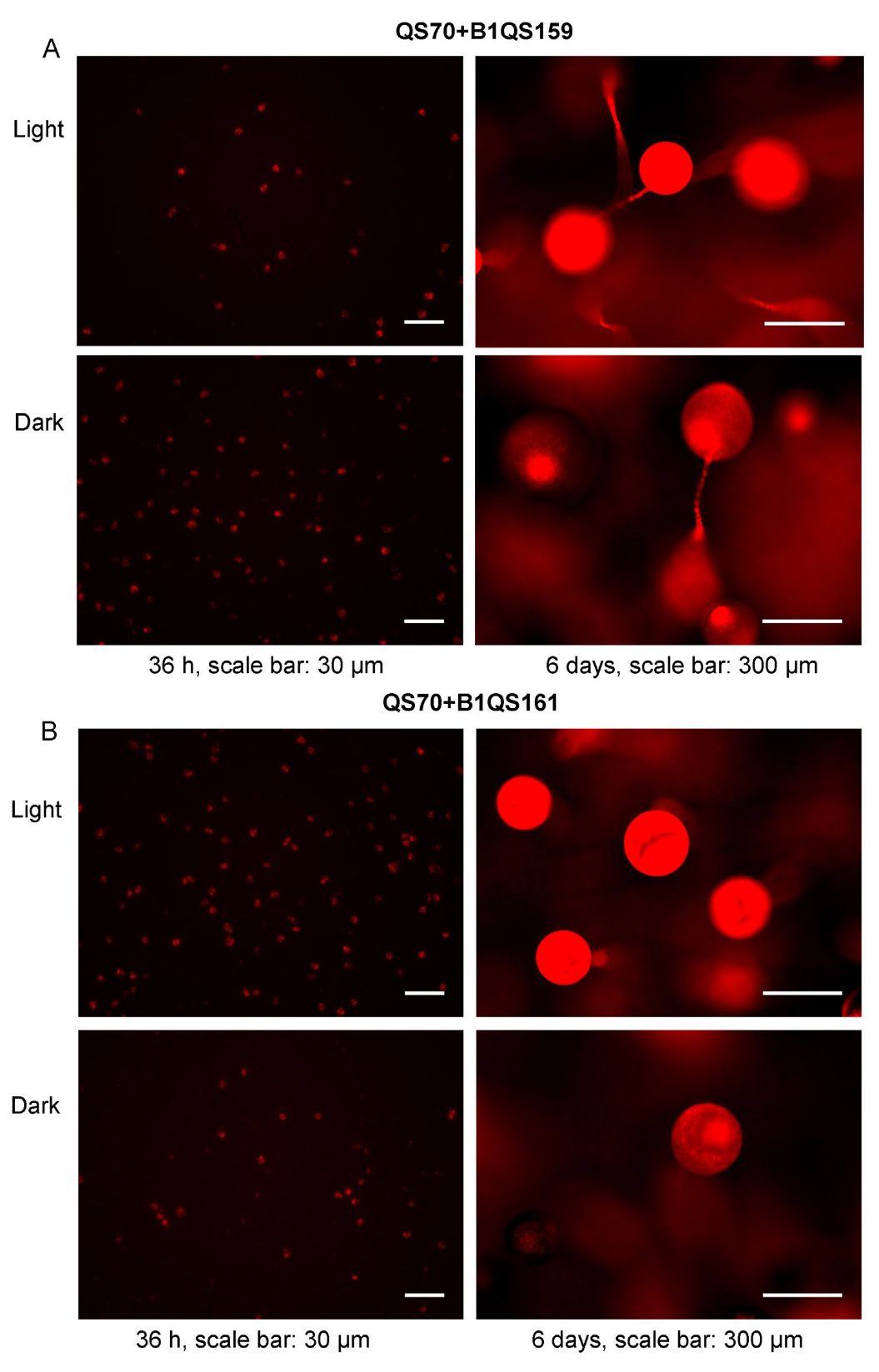


**Figure S10**. The fluorescence of two symbiotic bacteria B1QS159 and B1QS161 in amoeba QS70 vegetative and fruiting bodies under both light and dark treatments. Three biological replications were conducted in each pairing, demonstrating similar patterns in fluorescence detection.


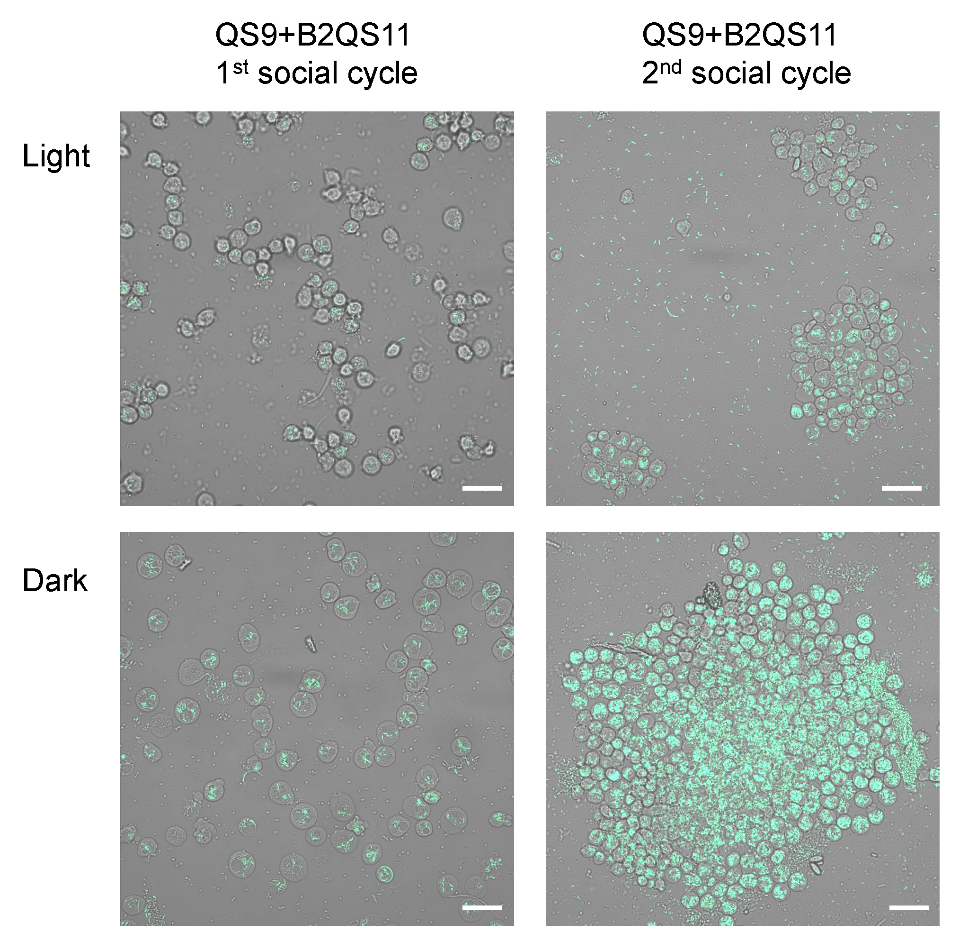


**Figure S11**. Fluorescence of amoeba QS9 cells at the exponential stage (incubated with *Paraburkholderia* B2QS11 for 36 h in the first social cycle and 42 h in the second social cycle) was measured under light and dark treatments across two social cycles. Three individual experiments were conducted in each pairing, showing similar trends in fluorescence changes (scale bar=20 μm).


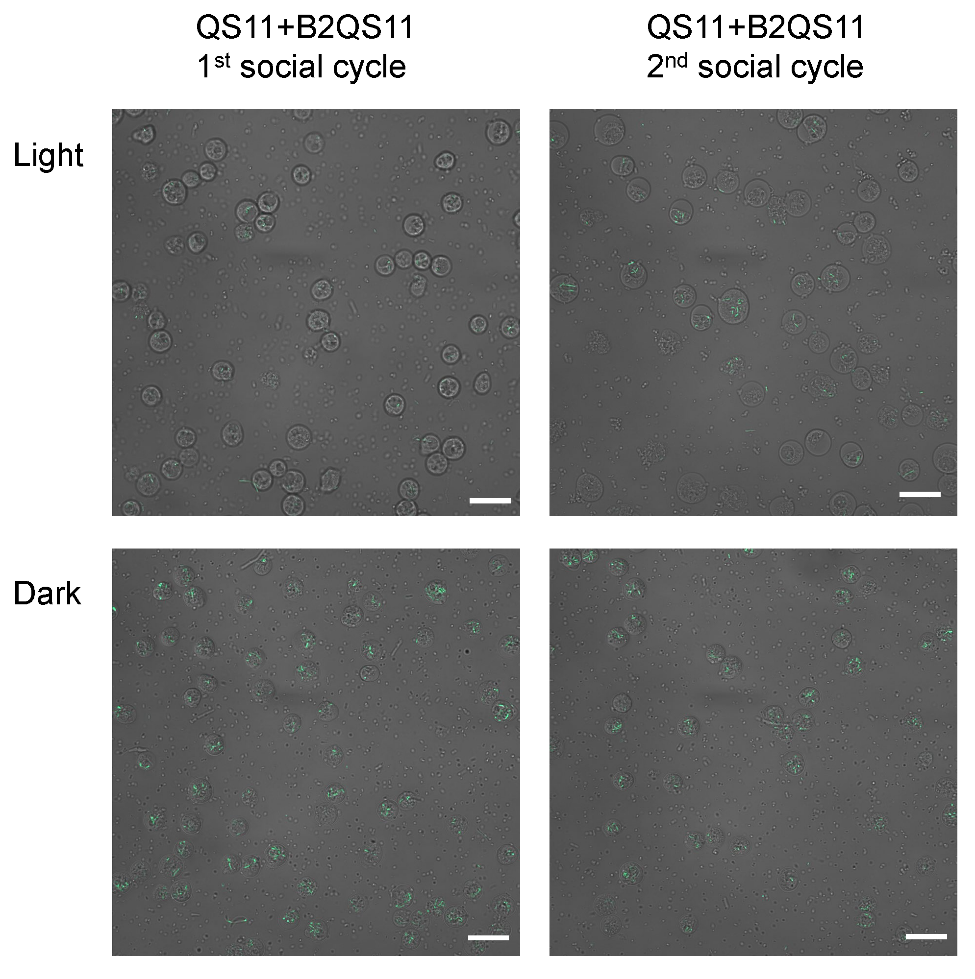


**Figure S12**. Fluorescence of amoeba QS11 cells at the exponential stage (incubated with *Paraburkholderia* B2QS11 for 36 h) was measured under light and dark treatments over two social cycles. Three individual experiments were performed in each pairing, indicating similar patterns in fluorescence changes, scale bar=20 μm).


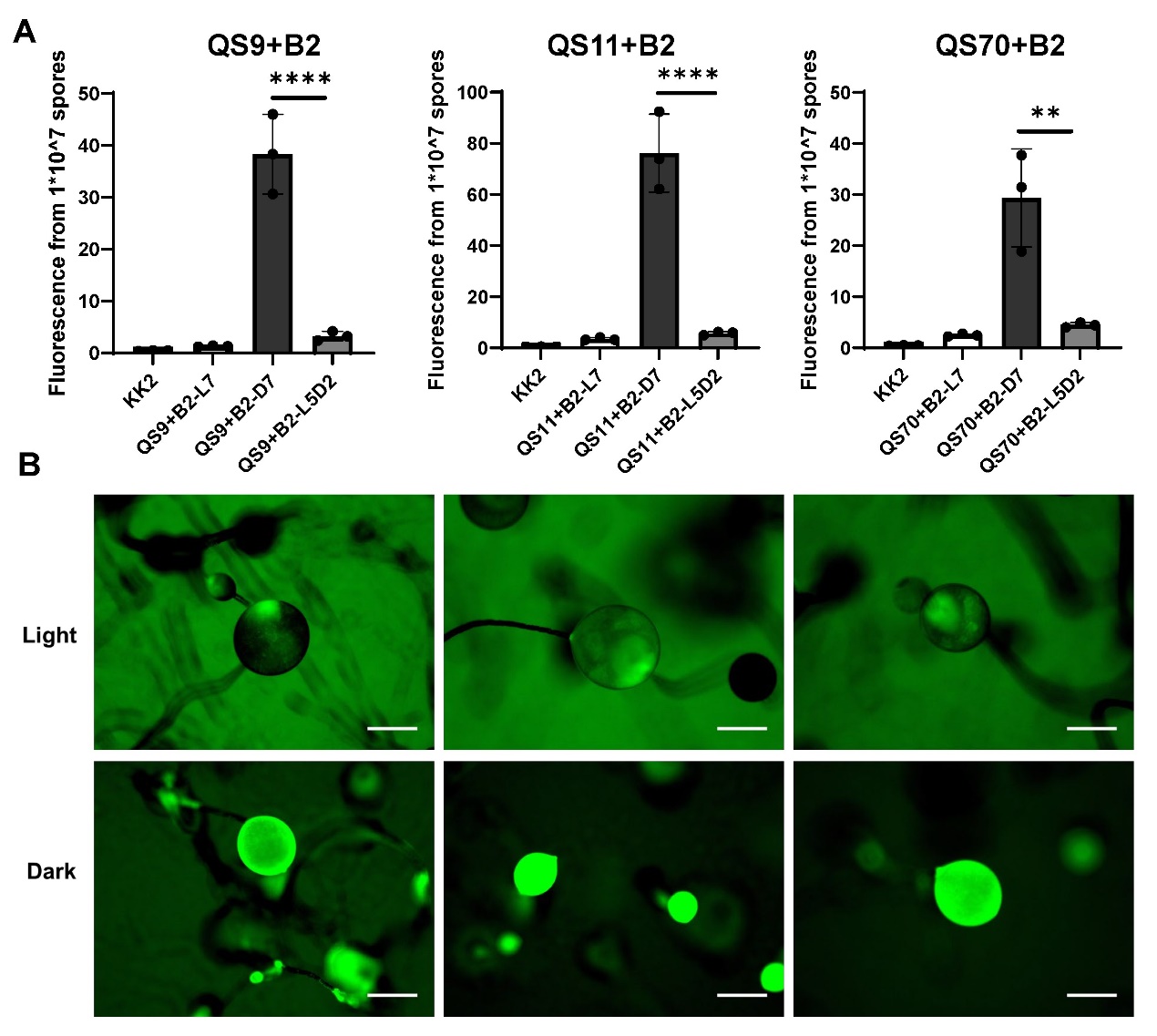


**Figure S13.** Fluorescence of amoeba fruiting body (incubated with *Paraburkholderia* B2QS11 for 7 days or as indicated). (A) Fluorescence measurement in certain amount of new germinated spores (QS9, QS11, QS70, 1×10^7^) with symbiotic B2QS11 under dark and light (Three biological replications were performed, n = 3; all error bars represent the SD). Asterisks indicate significance (**, P < 0.01; ****, P < 0.0001) according to the one-way ANOVA Tukey’s multiple-comparison test. L5D2 indicates 5 days light incubation and 2 days dark condition. (B) Fluorescence images of QS9, QS11, and QS70 fruiting body with symbiotic B2QS11 were taken under light and dark (scale bar=300 μm).


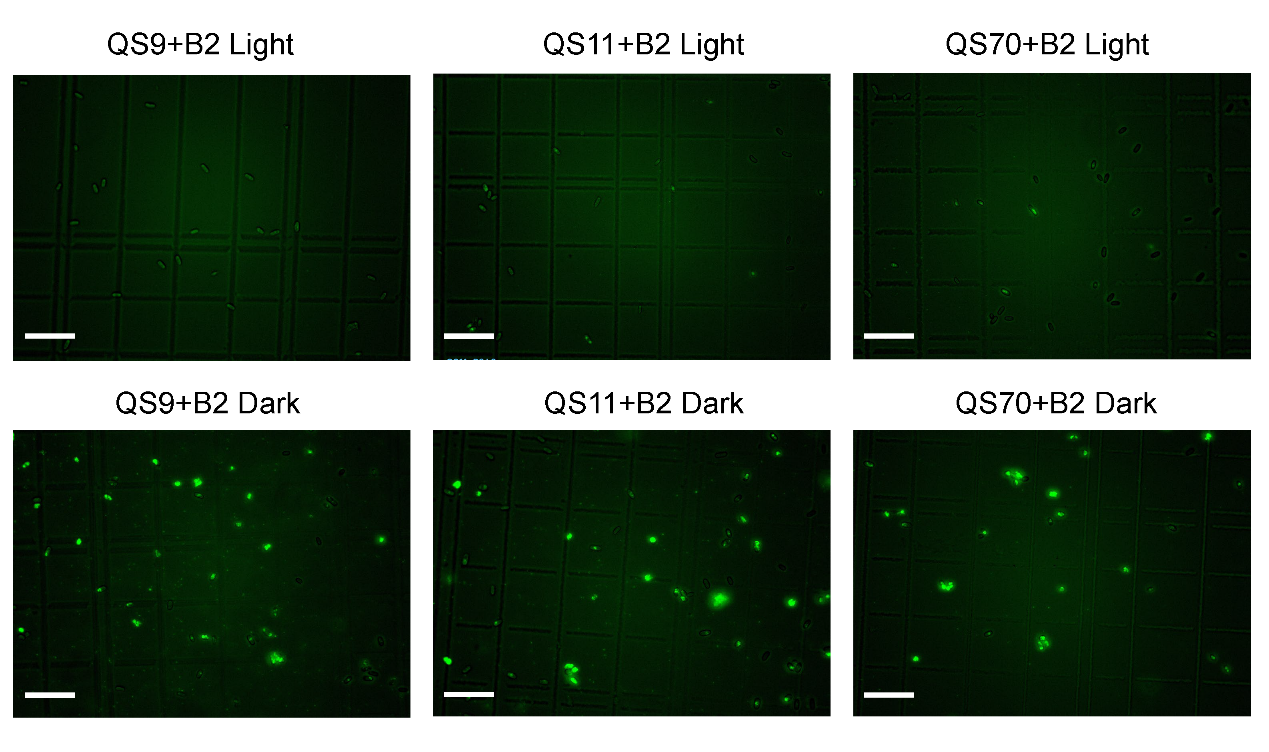


**Figure S14.** Intracellular B2QS11-GFP decreased in QS9, QS11 and QS70 amoeba spores under light illumination at 7 d. The fluorescence of QS9, QS11 and QS70 amoeba spores showed stronger fluorescence of symbiotic B2QS11-GFP under dark for 7 days (scale bar=40 μm).


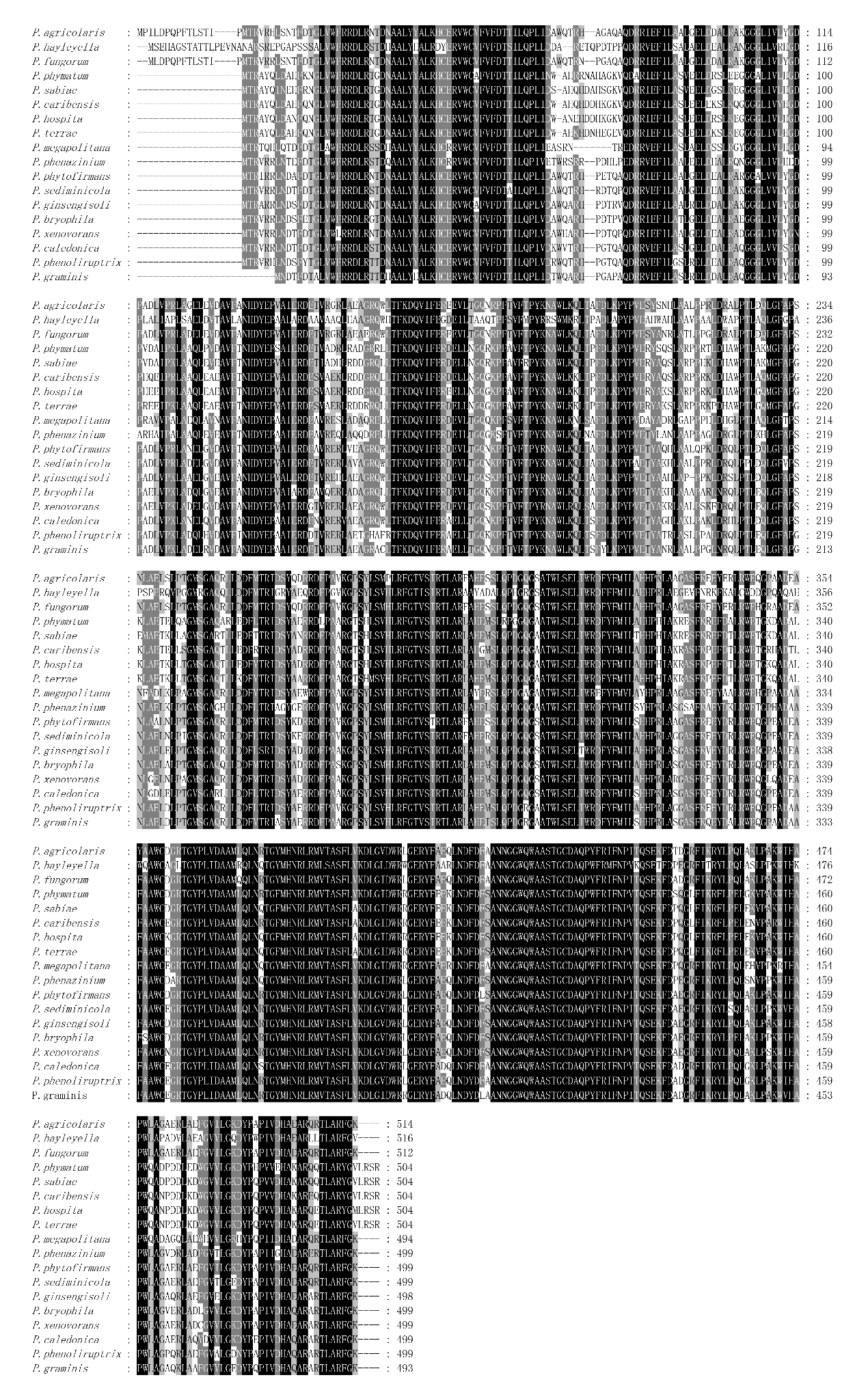


**Figure S15.** Alignment of blue-light photoreceptors cryptochrome/DNA photolyase in 18 *Paraburkholderia* species.

Based on JGI genome database, blue-light photoreceptors belonging to the family of cryptochrome/DNA photolyase were identified individually from selected 18 *Paraburkholderia* species genomes. Then those photoreceptors were aligned with high conserved protein sequences. B1QS70 belongs to *Paraburkholderia agricolaris*, while B2QS11 belongs to *Paraburkholderia hayleyella*. Other *Paraburkholderia* species were selected based on the phylogenetic tree with close evolutionary relationships to B1QS70 and B2QS11.
